# Computational design and evaluation of tacrine-based carbamate derivatives as acetylcholinesterase inhibitors

**DOI:** 10.1039/d6ra06329b

**Published:** 2026-08-26

**Authors:** Hajar El Ouarti, Abdelilah Toughzaoui, Kamal Laourdani, Abderrafie Drajou, Abdeslam Dakhama, Usama Raza, Mohammed Bensaka, Lamy M. M. Hamed, Khalil Azzaoui, Hammouti Belkheir, Ahmed El-Harairy, Kamal Moradi, Abdelkrim Ouammou

**Affiliations:** a Engineering Laboratory of Organometallic Molecular Materials and Environment, Faculty of Sciences, Sidi Mohamed Ben Abdellah University Fez Morocco hajar.elouarti@usmba.ac.ma; b Department of Pharmaceutical Chemistry, Dow College of Pharmacy, Dow University of Health Sciences Karachi Pakistan; c Department of Orthopedic Surgery, Hassan II University Hospital Center, Faculty of Medicine, Pharmacy and Dental Medicine, Sidi Mohamed Ben Abdellah University Fez Morocco; d Department of Environment and Agricultural Natural Resources, College of Agricultural and Food Sciences, King Faisal University Al-Ahsa Saudi Arabia lamy.hamed@kfu.edu.sa; e Euro-Mediterranean University of Fes Fes 30070 BP 15 Morocco; f Department of Chemical and Biomolecular Engineering, University of Nebraska-Lincoln Lincoln NE 68588 USA ael-harairy2@nebraska.edu; g Department of Soils and Water, Faculty of Agriculture, Damietta University New Damietta, Damietta 34517 Egypt

## Abstract

Alzheimer's disease is marked by a gradual decline in cognitive function accompanied by alterations in cholinergic signaling. Cholinergic function can be improved by targeting acetylcholinesterase (AChE). In this work, an *in silico* approach was applied to study a series of tacrine-based carbamate derivatives. 3D-QSAR models determined by CoMFA and CoMSIA and validated by cross-validation, external testing and Y-randomization were developed using a dataset of 27 compounds. A complementary 2D-QSAR model provided consistent results to support the proposed structure–activity relationships. The statistical parameters (*Q*^2^ > 0.67 and *R*^2^ > 0.93) show the influence of steric and hydrogen bond donor effects on activity. Then, four new derivatives were proposed and tested. The ADMET analysis revealed good pharmacokinetic properties and low predicted toxicity. The DFT calculations characterized the electronic properties of the designed derivatives, and the docking results showed stronger contacts within the active sites of TcAChE (2CKM) and hAChE (4EY7). The selected complexes with both AChE structures were stable in the molecular dynamics simulations for 200 ns. The binding energies and stable conformational behavior of the selected complexes were further confirmed by MM-GBSA, PCA, FEL and DCCM analyses. Overall, the results indicate that the designed derivatives could be promising candidates for AChE inhibition in Alzheimer's disease.

## Abstract 2

Integrated computational analyses identified tacrine-carbamate derivatives with favorable AChE binding and stable dynamic behavior, supporting their further experimental evaluation.

## Graphical abstract

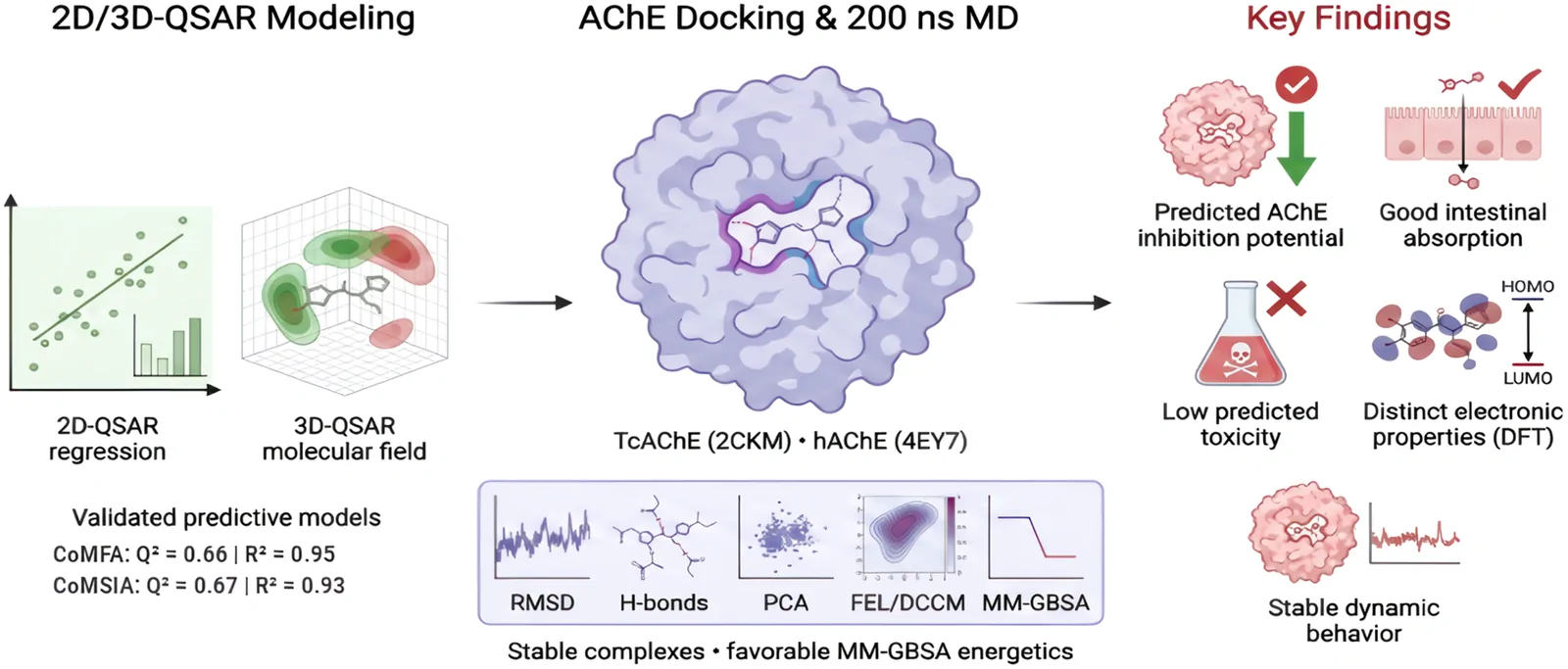

## 1. Introduction

Alzheimer's disease is a neurodegenerative disorder defined by progressive cognitive decline and impairment of the cholinergic system.^1^ Acetylcholinesterase (AChE) the enzyme that hydrolyzes acetylcholine, remains an important therapeutic target, since its inhibition increases the level of synaptic acetylcholine.^2^ Carbamate derivatives are interesting because of their structural analogy to acetylcholine and their ability to inhibit enzymes for a long time.^3^ Structural modifications have focused on the introduction of a tacrine moiety into carbamate derivatives in order to improve their activity. Although this compound was withdrawn from clinical use due to hepatotoxicity, it remains a well-known cholinesterase inhibitor and a valuable scaffold for structural optimization.^4^ Its association with carbamates has given rise to hybrid molecules exhibiting potent inhibitory activity and a long duration of action.^5^

**Fig. 1:**
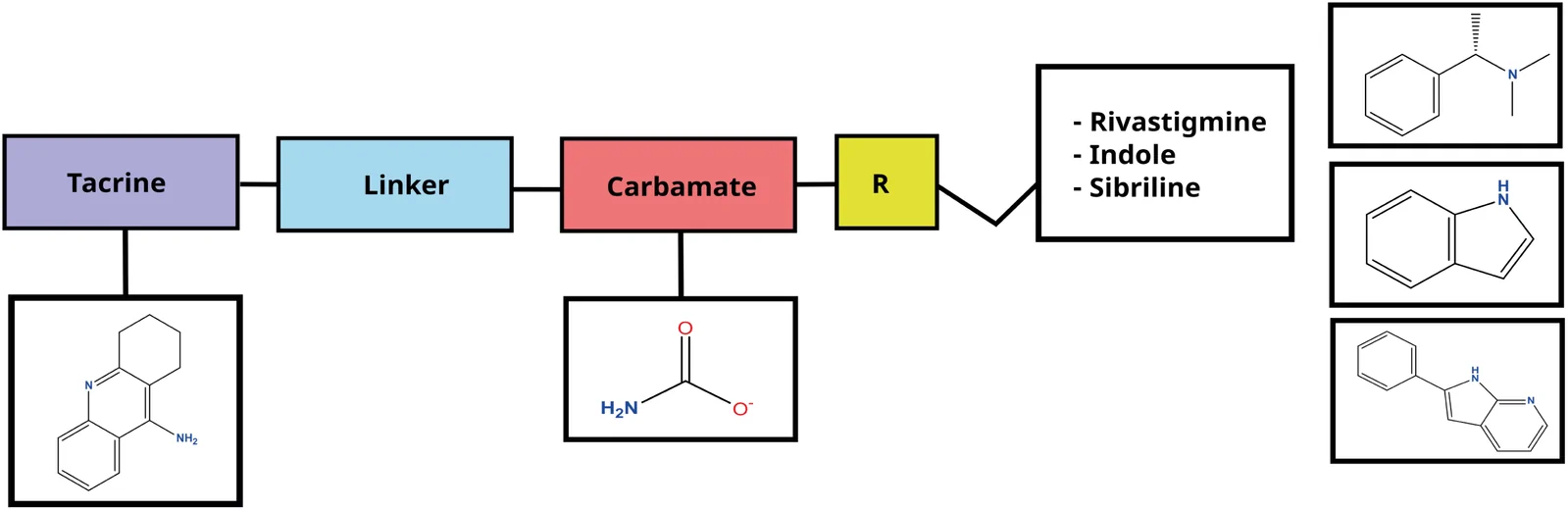
Chemical structures of tacrine-carbamate derivatives bearing different fragments (rivastigmine, indole, and sibriline).

Further optimization was accomplished by the introduction of pharmacologically relevant fragments, such as rivastigmine, indole and sibriline, chosen for their complementary properties (Fig. 1).^6^ Moreover, modification of the linker length and substitution pattern, namely the introduction of halogen groups on the tacrine ring, has been associated with enhanced activity and reduced toxicity.^7^ These developments led to the synthesis and experimental evaluation of a series of derivatives^4^ that are used here as the basis for the present study.

Drug discovery is greatly facilitated by computational methods, which allow for the rapid screening and analysis of potential compounds. Several complementary methods have been used in this work such as 3D-QSAR modeling, ADMET prediction, density functional theory (DFT), molecular docking and molecular dynamics simulations (MD). 3D-QSAR methods relate molecular descriptors with biological activity and allow activity prediction.^8^ ADMET analysis provides information about pharmacokinetic and toxicity profiles.^9^ DFT calculations are used to study the electronic properties of molecules.^10^ Molecular docking describes the binding interactions of ligands to their targets and molecular dynamics simulations serve to evaluate the time stability of the complexes.^11^

The present study is an attempt to screen tacrine based carbamate derivatives through these computational tools to identify the promising candidates, analyze their interaction with AChE and examine their predicted pharmacological properties.

## 2. Materials and methods

### 2.1 Data processing and structure optimization

**Fig. 2:**
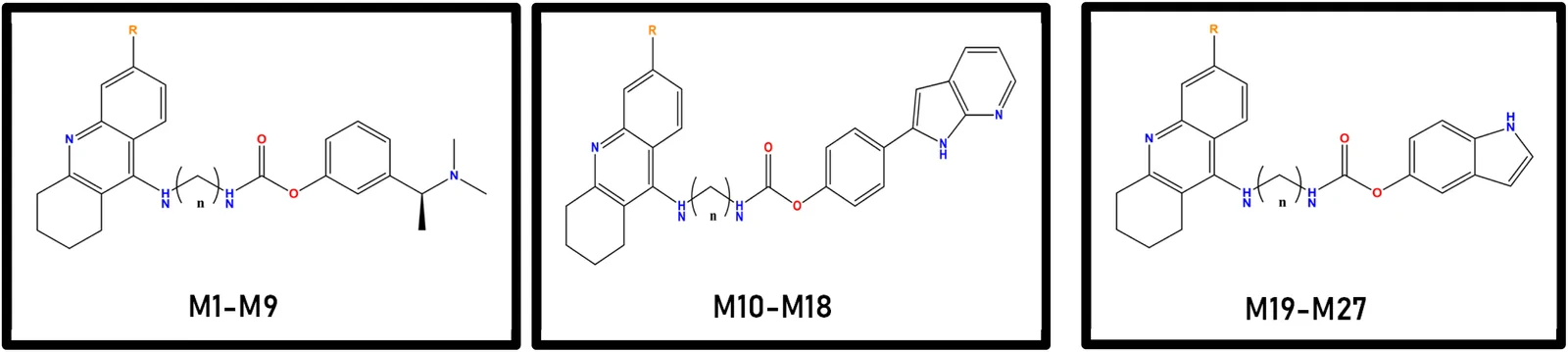
General chemical structure of tacrine-modified carbamates.

From literature.^4^ a collection of 27 carbamate compounds known to inhibit acetylcholinesterase was retrieved. The studied series was divided into two groups, consisting 21 molecules for model training and 6 molecules for external validation. The experimental IC_50_ values (nM) were converted to a logarithmic scale pIC_50_ = −log IC_50_ and were used as the output variable. Fig. 2 and Table 1 summarize the chemical structures with their activity values.

**Table 1:** AChE inhibitory activities of tacrine-modified carbamate derivatives

	Compound	R	*n*	AChE pIC_50_
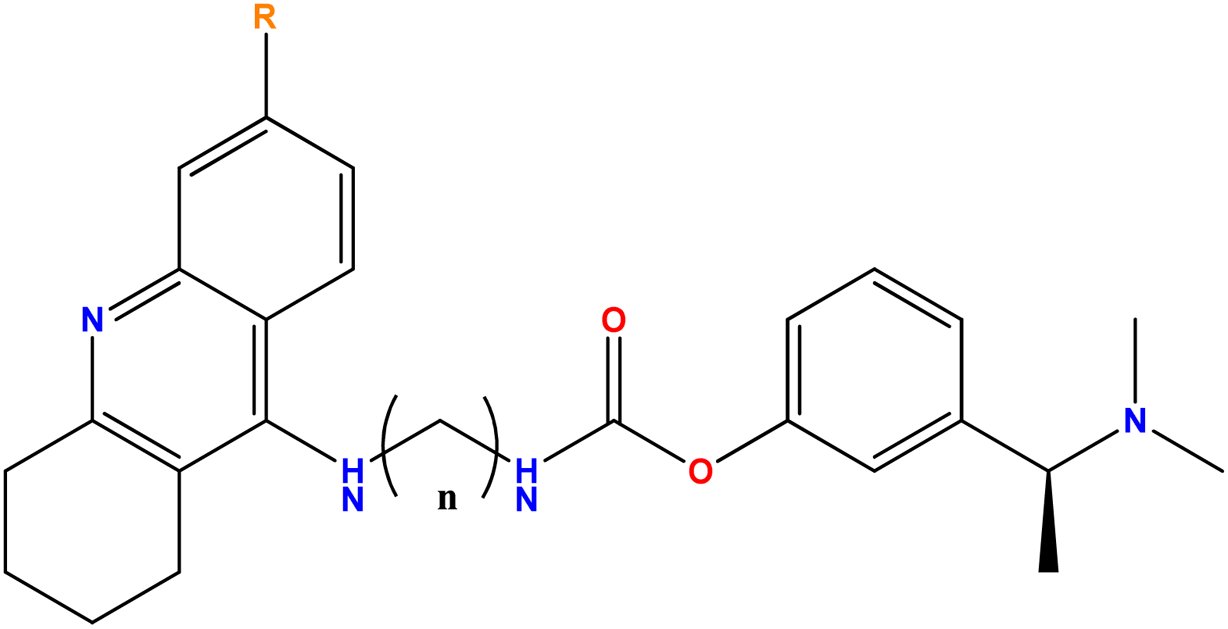	M1^a^	H	2	6.597
	M2	H	3	6.414
	M3	H	4	6.534
	M4^a^	H	5	6.931
	M5	F	5	7.422
	M6	F	6	7.375
	M7	Cl	5	8.081
	M8	Cl	6	7.591
	M9	Br	6	7.251
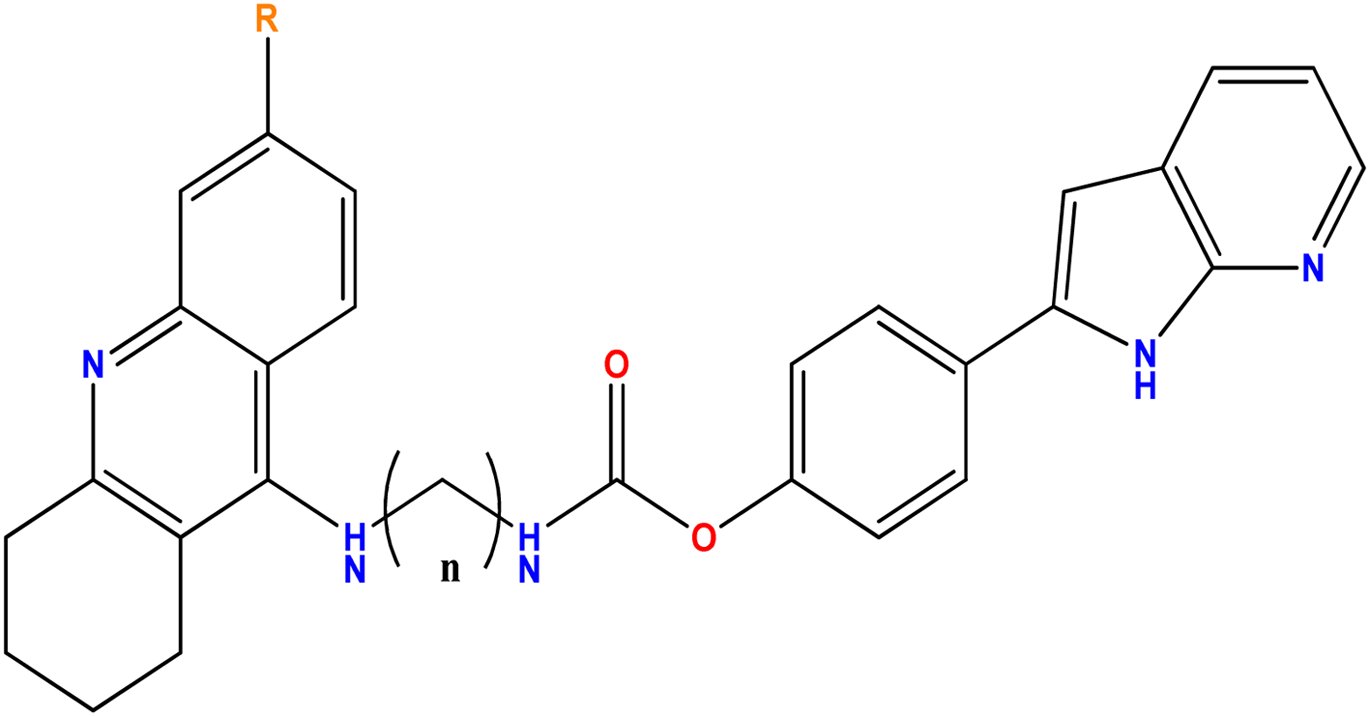	M10	H	3	6.633
	M11	H	4	6.688
	M12^a^	H	5	6.816
	M13	H	6	7.67
	M14	F	6	7.517
	M15	Cl	5	7.636
	M16^a^	Cl	6	7.189
	M17^a^	Br	5	7.228
	M18	Br	6	7.259
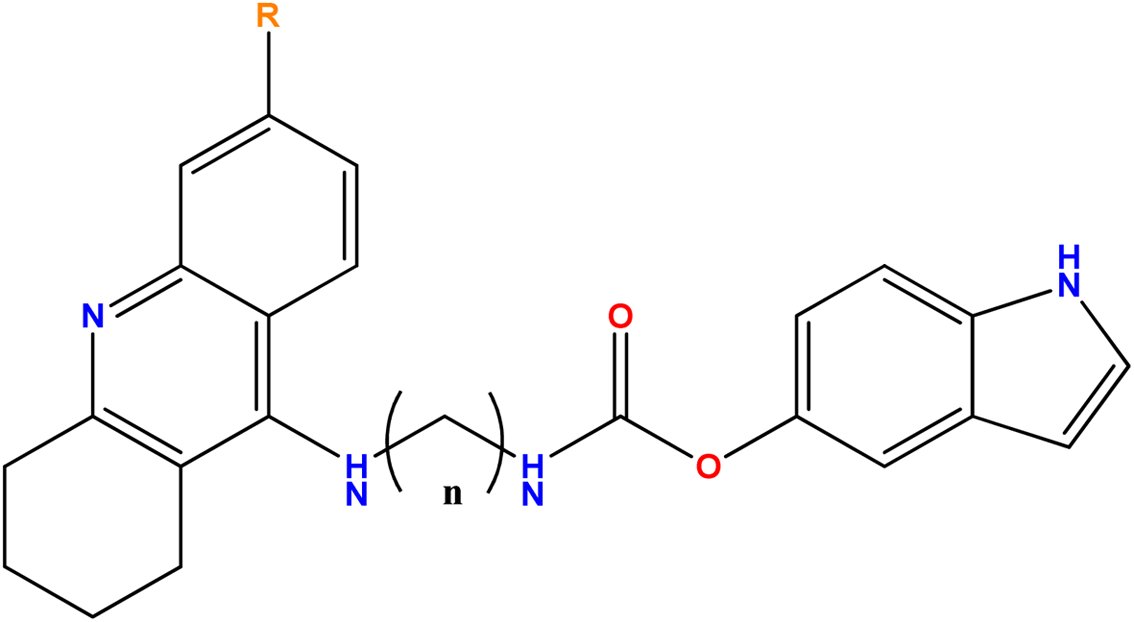	M19	H	2	5.901
	M20	H	3	6.258
	M21	H	4	6.292
	M22	H	5	6.375
	M23	F	5	6.563
	M24	Cl	5	6.606
	M25	Cl	6	6.816
	M26	Br	5	6.498
	M27^a^	Br	6	6.946

a Test set molecules; *n* = number of carbon atoms in the linker chain; *R* = substituent.

### 2.2 3D-QSAR

#### 2.2.1 Structure alignment

The alignment of molecules was considered to be an indispensable step for or the development of a validated 3D-QSAR model. The series studied includes 27 molecular structures that were optimized by the Tripos force field^12^ and using Gasteiger-Hückel partial charges.^13^

A convergence criterion of 0.001 kcal mol^−1^ and a maximum of 1000 iterations were applied during the optimization process, using the SYBYL-X 2.0 software.^14^ Chemical compounds were aligned through Distill rigid alignment module.

**Fig. 3:**
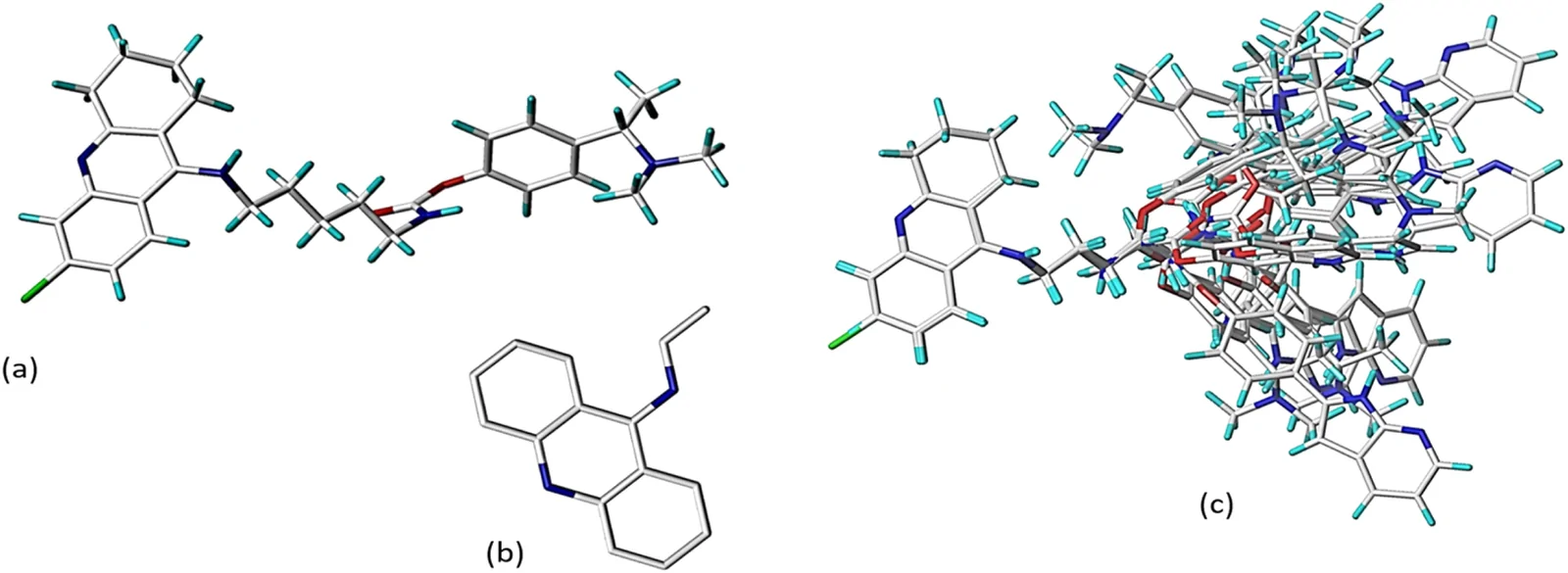
Alignment and superposition of the dataset using compound M7 as the reference model: (a) Reference structure, (b) common core, (c) set of aligned molecules.

The compound M7 was identified to be the most active molecule with a pIC_50_ value of 8.081, and was selected as the reference structure to guide the superposition of the remaining compounds, as illustrated in Fig. 3. In ligand-based 3D-QSAR, usually the most active compound is used as a template for alignment, since it is assumed to represent the most favorable interaction pattern with the biological target.

The choice provides a consistent structural background for the development of reliable QSAR models.

#### 2.2.2 Descriptor calculation

The structural determinants of biological activity were studied using the 3D-QSAR models constructed on CoMFA^15^ and CoMSIA.^16^ These approaches consisted in the evaluation of the steric, electrostatic, hydrophobic and hydrogen-bonding contributions.

The CoMFA model was constructed from the training set using PLS regression.^17^ Steric and electrostatic fields were calculated with the Tripos force field^12^ on a 3D grid with a distance-dependent dielectric constant. Grid spacing ranged from 0.5 to 3.0 Å, and a cutoff of 30 kcal mol^−1^ was applied.^18^ Model validation was performed using LOO cross-validation to estimate *Q*^2^ and determine the optimal number of components.^19^ Final model statistics (*R*^2^, SEE, *F*) were generated *via* non-cross-validated analysis.^20^ CoMSIA analysis was conducted with similar settings to explore steric, electrostatic, hydrophobic and hydrogen-bond contributions.

### 2.3 2D-QSAR

To improve the reliability of the constructed 3D-QSAR model, a complementary 2D-QSAR approach based on machine learning was performed using the same dataset and the same division into training (21 compounds) and test (6 compounds) sets.^4^ Three molecular descriptors (SMR, NumHeteroAtoms, and FractionCSP3) were selected based on their physicochemical relevance and their complementary contribution to describing the structure–activity relationship, and were normalized to ensure that all variables were on a comparable scale prior to model construction. A regression model was then built using the training set and evaluated on the external test set using the coefficient of determination *R*_test_^2^, to evaluate the predictive capacity of the model.^21^ Internal validation of the model was carried out using a *k*-fold cross-validation procedure, which was used to estimate the cross-validated coefficient (*Q*^2^), providing insight into the stability and robustness of the model.^18^

### 2.4 Y-randomization

As an additional validation step, Y-randomization was employed to verify the model resilience. This is done *via* random permutation of the pIC_50_ values and rebuilding the model after each permutation. The reduced *Q*^2^ and *R*^2^ values in comparison with the original model suggest that there is no chance link. Model reliability was further evaluated using the *cR*_p_^2^ parameter, calculated according to eqn (1), where 
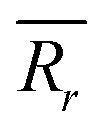
 denotes the mean correlation coefficient derived from the randomized models. This parameter compares the correlation of the original model with those derived from randomized models. A value higher than 0.5 indicates a robust model.^22^1
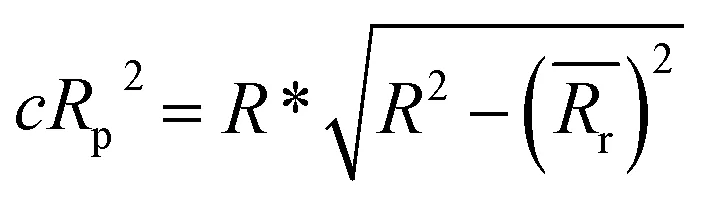


### 2.5 Bootstrap validation

To further evaluate the robustness and reliability of the developed CoMSIA model, a bootstrap validation^23^ procedure was carried out using SYBYL-X 2.1. We performed a total of 1000 bootstrap iterations fixing the optimal number of components to four corresponding to the final CoMSIA model. In this procedure, a number of resampled training subsets were automatically generated from the original data set to evaluate the reproducibility of the statistical parameters and the stability of the selected molecular field contributions. In addition, this method made possible the estimation of the possible statistical bias and the evaluation of the overall confidence of the developed model by comparing the statistical parameters obtained from the original data set with those generated during the bootstrap resampling process. Therefore, this validation approach was used to study the reproducibility, robustness and statistical stability of the proposed 3D-QSAR model.

### 2.6 Model applicability

A critical step in the validation of a 3D-QSAR model is the assessment of the applicability domain, which defines the chemical domain where the model's predictions are considered to be reliable.^24^ To identify atypical or influential compounds, the leverage method was employed.^25^ This method is centered on the calculation of leverage values (*h*) for each compound, using the matrix of descriptors employed in the model construction. The leverage values (*h*_*i*_) as well as the critical leverage value (h*), delineating the boundary of the applicability domain, were calculated according to eqn (2) and (3). where *x*_*i*_ is the descriptor vector of compound *i*, *X* is the matrix of the *k* descriptor values used to build the model and *n* is the total number of compounds in the training.^25^

2 *h*_*i*_ = *x*_*i*_^*T*^(*X*^*T*^*X*)^−1^*x*_*i*_

3
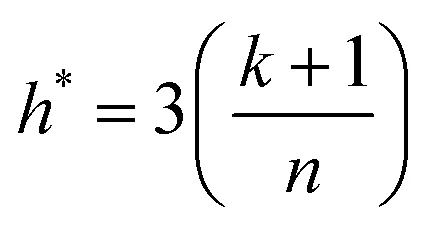


### 2.7 ADMET profiling

ADMET profiling was considered a key approach widely used in molecular modeling to examine pharmacokinetic and toxicological properties of compounds.^26^ This methodology is widely applied in the initial phases of drug discovery, since it offers trustworthy information about the pharmacokinetic profile of candidate molecules.

In this study, ADMET profile of the designed molecules was predicted using the pkCSM online platform,^27^ and synthetic accessibility (SA) and Medicinal Chemistry Evolution (MCE-18) were obtained from ADMETlab 3.0.^28^ The results obtained were correlated with those of molecule M7, identified as the most active reference compound in the 3D-QSAR series for comparative validation purposes. To assess the solubility behavior, the aqueous solubility was expressed as log mol L^−1^. Intestinal permeability was evaluated using the Caco-2 cell model and human intestinal absorption (HIA) was considered to estimate oral absorption. Distribution properties were evaluated by blood–brain barrier permeability *via* the log BB descriptor.^29^

The metabolic behavior was examined by prediction of the interaction with the main cytochrome P450 isoenzymes CYP1A2, CYP2C9, CYP2C19, CYP2D6 and CYP3A4 when the classification of the compounds as substrates or inhibitors.^25^ Excretion characteristics were assessed by total clearance values (log mL min^−1^ kg^−1^), which are a measure of the overall hepatic and renal clearance rate of the compounds.^25^ The toxicological risk assessment included prediction of AMES mutagenicity, hERG channel inhibition, maximum tolerated dose and skin sensitization. Preliminary analysis was performed to confirm the expected pharmacokinetics and safety of the designed compounds before proceeding to further studies.

### 2.8 DFT analysis

DFT is a powerful computational tool for studying the electronic properties of chemical compounds and is an important tool in rational drug design, allowing the evaluation of molecular electronic structure.^30^

In this work DFT calculations were performed on four carbamate derivatives proposed as acetylcholinesterase inhibitors and on the molecule M7 used as reference compound. The geometrical optimization and vibrational frequencies calculations were performed with Gaussian 09.^31^ The graphical interface used was GaussView 5.0.8.^32^ All calculations were performed in the gas phase with the hybrid B3LYP functional.^33^M7 and N1–N4 were considered in their neutral singlet states, with a total charge of 0 and a multiplicity of 1. The neutral amino form of the tacrine moiety and the canonical tautomeric forms represented in the molecular structures were retained throughout the calculations, without generating alternative tautomers. In the calculations the 6-31G(d,p) basis set was used for non-metal atoms (C, N, O, H and F) and the SDD basis set for bromine and iodine atoms.^34^ Vibrational frequencies were calculated to confirm the optimized structures as true minima on the potential energy surface, which was confirmed by the absence of imaginary frequencies.^35^ The HOMO and LUMO energies were determined from the optimized geometries and then the electronic gap which is the difference in energy between these orbitals (eqn (4)).^36^ Furthermore, some global reactivity descriptors including ionization energy (*I*), electron affinity (*A*), hardness (*η*), softness (*σ*), chemical potential (*µ*), electronegativity (*χ*) and the global electrophilicity index (*ω*) were calculated using the standard equations^35^ from the HOMO and LUMO energies in order to characterize the electronic properties of the studied compounds. Finally, the HOMO and LUMO orbitals and molecular electrostatic potential (ESP) maps were visualized in order to identify the prone regions to the electrophilic or nucleophilic interactions, in accordance with the conceptual DFT theory.^36^

4 Electronic gap (*E*_g_): *E*_g_ = *E*_LUMO_ − *E*_HOMO_

5 Ionization energy (*I*): *I* = −*E*_HOMO_

6 Electron affinity (*A*): *A* = −*E*_LUMO_

7
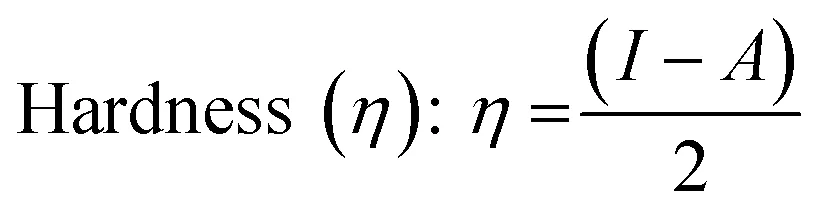
8
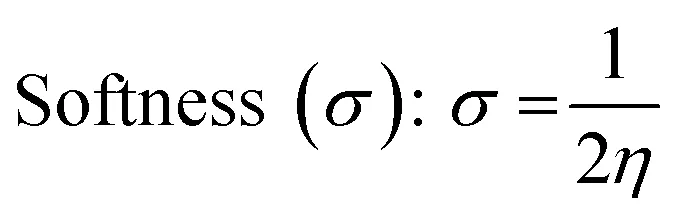
9
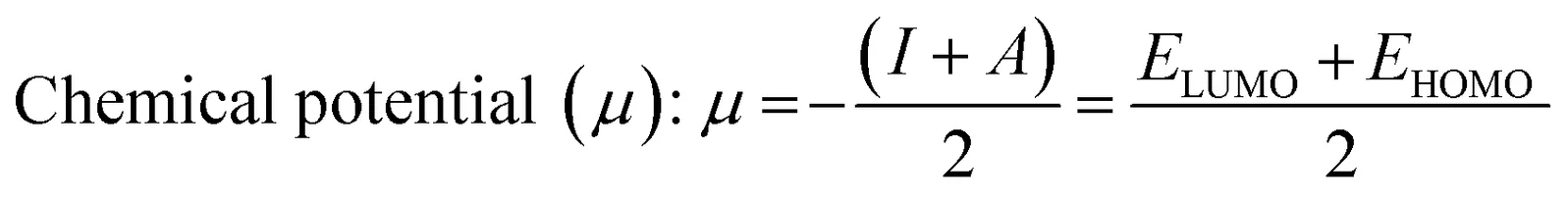

10 Electronegativity (*χ*): *χ* = −*µ*

11
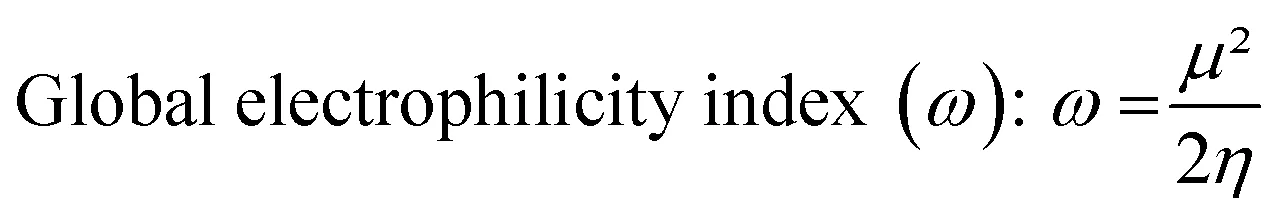


### 2.9 Docking analysis

Molecular docking is a technique for predicting the ligand binding modes at the active site of a protein and assessing binding affinity and intermolecular interactions.^11^ The ligand–receptor interactions were studied using molecular docking simulations with the program AutoDock 4.2.^37^

The crystallographic structures of *Torpedo californica* acetylcholinesterase (TcAChE; PDB ID: 2CKM, resolution 2.15 Å) and human acetylcholinesterase (hAChE; PDB ID: 4EY7, resolution 2.35 Å) were obtained from the Protein Data Bank.^38^ The 2CKM structure was retained for comparison with the reference study, whereas 4EY7 was included to evaluate the designed compounds against human AChE and to ensure consistency with the enzyme used in the experimental biological activity data. Co-crystallized ligands and all water molecules were removed with BIOVIA Discovery Studio Visualizer 2025.^39^ The receptors and ligands were prepared with AutoDockTools version 1.5.7.^37^ Polar hydrogen atoms and Kollman charges were assigned to the receptors, whereas polar hydrogen atoms and Gasteiger partial charges were assigned to the ligands before conversion to PDBQT format.^40^M7 and N1–N4 were docked in their neutral forms, with a net charge of 0. The neutral amino form of the tacrine moiety and the canonical tautomeric forms shown in the molecular structures were retained, and no alternative tautomers were generated during ligand preparation. For the TcAChE structure 2CKM, the grid box was centered at the enzyme active site (*x* = 0.603, *y* = 64.680, *z* = 68.925) with a size of 60 × 60 × 60 grid points and a spacing of 0.375 Å. For the human AChE structure 4EY7, the grid box was centered on the active-site region defined by the co-crystallized ligand at *x* = −14.108, *y* = −43.833, and *z* = 27.670, using the same grid dimensions of 60 × 60 × 60 points and a spacing of 0.375 Å. The resulting complexes were analyzed to identify the key interactions involved in ligand binding and to compare the binding behavior of the designed compounds within the TcAChE and hAChE active sites.

### 2.10 Molecular dynamics

Docking studies provide an early estimate for ligand–target interactions but are limited to their static nature. This limitation is overcome by molecular dynamics (MD) simulations, which allow the evaluation of complex stability and conformational changes in an explicitly solvated system under controlled conditions.^41^ Molecular dynamics simulations of 200 ns were performed for each system to study the dynamic behavior of *Torpedo californica* acetylcholinesterase (TcAChE, PDB ID: 2CKM) and human acetylcholinesterase (hAChE, PDB ID: 4EY7), each separately complexed with M7, N2, N3, and N4. The selected ligands were simulated in their neutral forms, each with a formal charge of 0. The canonical tautomeric forms present in the initial ligand structures were retained throughout system preparation and simulation.

The MD simulations were conducted using Desmond (Schrödinger LLC) with the OPLS_2005 force field.^42^ Ligand atom types and partial atomic charges were assigned during system preparation according to the OPLS_2005 force–field parameters. The prepared systems were further solvated in an orthorhombic simulation box with TIP3P water molecules at a buffer distance of 10 Å from the solute. A nominal NaCl concentration of approximately 50 mM was used, with additional counterions added as required to ensure overall charge neutrality. No separate conventional energy-minimization stage was included in the recorded workflow. Before the production simulations, all 2CKM and 4EY7 complexes underwent the same 160 ps Desmond relaxation and equilibration protocol, comprising restrained Brownian dynamics followed by restrained NVT/NPT stages and a final unrestrained NPT stage at 310 K. Restraints were applied to the solute heavy atoms during the initial stages and removed before production.

The equations of motion were integrated using the r-RESPA scheme, with time steps of 2 fs for the two inner levels and 6 fs for the outer level during the production simulations. Long-range electrostatic interactions were treated using the Particle Mesh Ewald method with a cutoff distance of 9.0 Å. The simulations were conducted under NPT conditions at 310 K and 1.01325 bar. Temperature was controlled using a Nose–Hoover chain thermostat with a relaxation time of 1.0 ps, while pressure was maintained using the Martyna-Tobias-Klein barostat with a relaxation time of 2.0 ps.^43^ No positional restraints were applied during the production simulations. Trajectory frames were saved at intervals of 200 ps. After the simulations, RMSD and RMSF were analyzed using the Simulation Interaction Diagram module implemented in Desmond. Protein–ligand hydrogen bonds were defined using a donor–acceptor distance cutoff of 2.5 Å, a minimum donor angle of 120°, and a minimum acceptor angle of 90°.

The binding free energies (Δ*G*_bind_) of the complexes were estimated using the MM-GBSA method implemented in the Schrödinger Prime module. The binding free energy was calculated according to eqn (12), where *G*_complex_, *G*_protein_, and *G*_ligand_ represent the free energies of the complex, protein, and ligand, respectively.^25^

The dynamic behavior of the studied complexes was characterized by principal component analysis (PCA), dynamic cross-correlation matrix (DCCM) analysis, and free energy landscape (FEL) analysis. The dominant motions during the simulation were identified using PCA. DCCM analysis provided insight into correlated and anti-correlated motions of the residues. FEL analysis was used to identify the most energetically favorable conformational states explored by the complexes.

12 Δ*G*_bind_ = *G*_complex_ − (*G*_protein_ + *G*_ligand_)

## 3. Results and discussion

### 3.1 3D-QSAR model validation

The 3D-QSAR models were constructed using the CoMFA and CoMSIA methodologies to correlate molecular descriptors with AChE inhibition activity expressed as pIC_50_. Tables 2–4 presents the statistical parameters, the estimated and measured values and residuals.

**Table 2:** Statistical parameters and field contributions of the developed CoMFA and CoMSIA models^a^

Field contributions											
	*Q* ^2^	*R* ^2^	*R*_pred_^2^	SEE	*F*	*N*	S	E	H	D	A
CoMFA	0.665	0.948	0.957	0.151	73.39	4	0.436	0.564	—	—	—
CoMSIA	0.672	0.932	0.890	0.173	54.927	4	0.296	—	—	0.704	—

a *Q* ^2^ corresponds to the cross-validated correlation coefficient, while *R*^2^ represents the conventional correlation coefficient and *R*_pred_^2^ indicates the predictive ability based on the external test set. SEE refers to the standard error of estimation and *F* denotes the Fisher statistic. *N* represents the number of components used in the model. Field contributions include steric, electrostatic, hydrophobic, hydrogen-bond donor, and hydrogen-bond acceptor effects.

The CoMFA model exhibited high predictive ability (*Q*^2^ = 0.665, *R*^2^ = 0.948, *R*_pred_^2^ = 0.957) with steric and electrostatic contributions of 43.6% and 56.4%, respectively. This indicates that modulation of activity depends not only on the molecular size but also on the charge distribution. The CoMSIA model also showed good behavior (*Q*^2^ = 0.672, *R*^2^ = 0.932, *R*_pred_^2^ = 0.890) with the major contribution from the steric and hydrogen-bond donor fields.

**Fig. 4:**
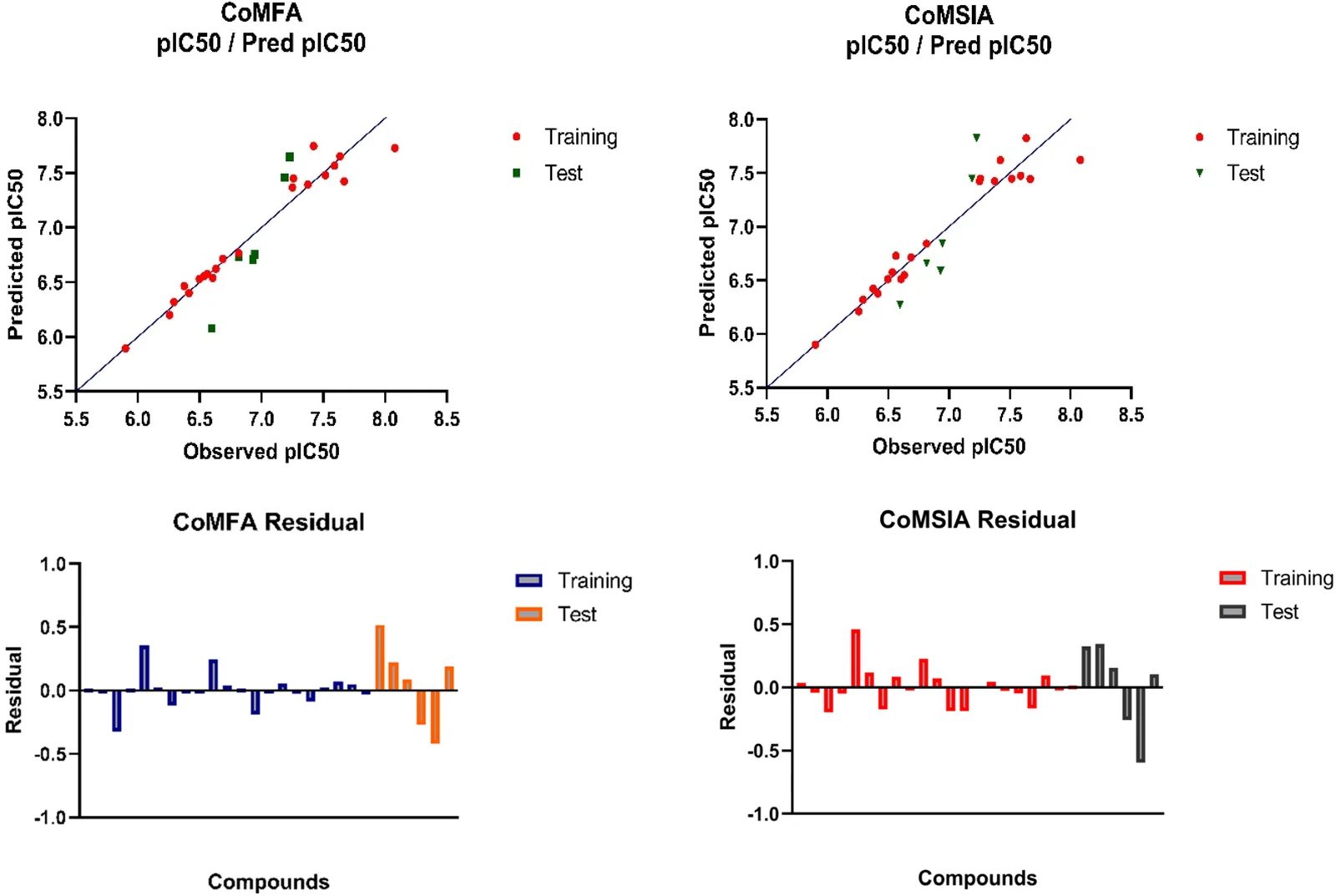
Correlation between experimental and predicted pIC_50_ values and residual distribution for the CoMFA and CoMSIA models.

The analysis of the CoMSIA field combinations (Table 3) suggested that the steric and hydrogen-bond donor model gave the best predictive performance, which highlights the combined impact of these descriptors in describing structure–activity relationships. The low residuals and the agreement between estimated and measured pIC_50_ values shown in Table 4 indicate that the models are consistent for both training and test sets. The plots shown in Fig. 4 indicate a good agreement between the calculated and observed pIC_50_ values. The corresponding residual distributions are generally close to zero without any clear systematic trends, indicating that there is no significant bias in the predictions. Overall, the results suggest that the developed models are stable and predictive and can be used to aid the design of new AChE inhibitors.

**Table 3:** Relative field contributions in the CoMSIA models^a^

S	E	H	D	A	*Q* ^2^	*R* ^2^	S	E	H	D	A	*Q* ^2^	*R* ^2^
**+**	**+**	**+**	**+**	**+**	0.609	0.943	**+**		**+**		**+**	0.567	0.939
**+**	**+**	**+**	**+**		0.627	0.946		**+**	**+**		**+**	0.507	0.950
**+**	**+**	**+**		**+**	0.576	0.947		**+**		**+**	**+**	0.601	0.940
**+**	**+**		**+**	**+**	0.638	0.939	**+**	**+**				0.650	0.947
**+**		**+**	**+**	**+**	0.620	0.938	**+**		**+**			0.642	0.928
	**+**	**+**	**+**	**+**	0.573	0.944		**+**	**+**			0.534	0.952
**+**	**+**	**+**			0.601	0.950	**+**				**+**	0.580	0.895
**+**	**+**			**+**	0.612	0.940		**+**			**+**	0.520	0.931
**+**			**+**	**+**	0.639	0.923		**+**		**+**		0.601	0.945
		**+**	**+**	**+**	0.567	0.936	**+**			**+**		0.672	0.932
**+**	**+**		**+**		0.653	0.943				**+**	**+**	0.562	0.916
**+**		**+**	**+**		0.652	0.943			**+**	**+**		0.596	0.923
	**+**	**+**	**+**		0.585	0.948			**+**		**+**	0.456	0.937

a Field contributions include steric, electrostatic, hydrophobic, hydrogen-bond donor, and hydrogen-bond acceptor effects. *Q*^2^ is the cross-validated correlation coefficient, *R*^2^ is the conventional correlation coefficient. (+) indicates included CoMSIA fields.

**Table 4:** Experimental and predicted pIC_50_ values and residuals of the studied compounds

Compound	AChE pIC_50_	CoMFA		CoMSIA	
		Predicted pIC_50_	Residuals	Predicted pIC_50_	Residuals
M2	6.414	6.400	0.014	6.377	0.037
M3	6.534	6.555	−0.021	6.577	−0.043
M5	7.422	7.747	−0.325	7.620	−0.198
M6	7.375	7.392	−0.017	7.424	−0.049
M7	8.081	7.729	0.352	7.621	0.460
M8	7.591	7.567	0.024	7.474	0.117
M9	7.251	7.367	−0.116	7.425	−0.174
M10	6.633	6.624	0.009	6.549	0.084
M11	6.688	6.713	−0.025	6.713	−0.025
M13	7.670	7.425	0.245	7.445	0.225
M14	7.517	7.478	0.039	7.446	0.071
M15	7.636	7.653	−0.017	7.824	−0.188
M18	7.259	7.450	−0.191	7.447	−0.188
M19	5.901	5.895	0.006	5.901	0
M20	6.258	6.201	0.057	6.212	0.046
M21	6.292	6.319	−0.027	6.32	−0.028
M22	6.375	6.465	−0.090	6.422	−0.047
M23	6.563	6.573	−0.010	6.729	−0.166
M24	6.606	6.539	0.067	6.511	0.095
M25	6.816	6.766	0.050	6.841	−0.025
M26	6.498	6.531	−0.033	6.512	−0.014
M1^a^	6.597	6.080	0.517	6.272	0.325
M4^a^	6.931	6.708	0.223	6.586	0.345
M12^a^	6.816	6.731	0.085	6.66	0.156
M16^a^	7.189	7.458	−0.269	7.447	−0.258
M17^a^	7.228	7.646	−0.418	7.824	−0.596
M27^a^	6.946	6.756	0.190	6.842	0.104

a Test set molecules.

### 3.2 3D molecular field maps

**Fig. 5:**
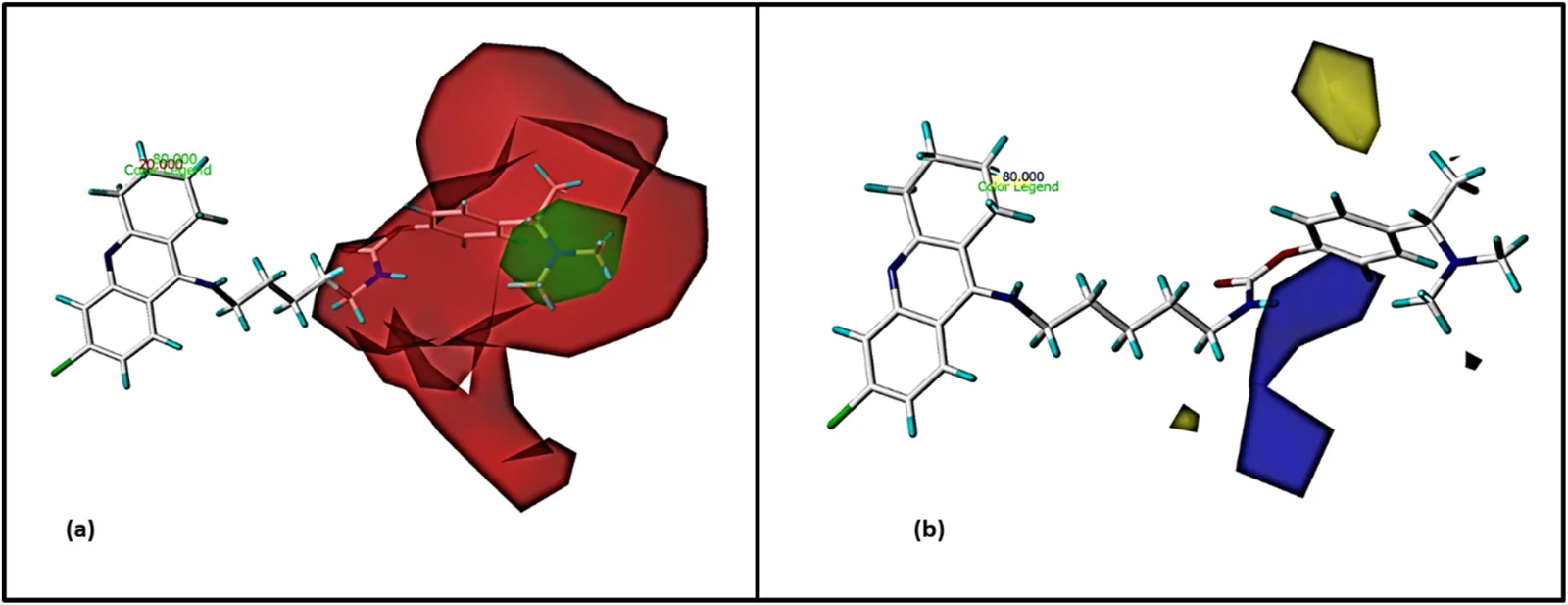
CoMSIA 3D contour maps illustrating interaction regions around the most active compound. (a) Steric field (green: favored; red: disfavored) and (b) donor field (blue: favored; yellow: disfavored).

The interpretation of the three-dimensional contour maps of the CoMSIA model obtained for the most active molecule (Fig. 5) emphasizes the most important spatial regions controlling the biological activity. The steric contour map indicates a significant red region around the center portion of the molecule indicating that excessive steric bulk in this location is not advantageous for biological activity. In contrast, a small green area surrounding the aromatic ring implies that a slight increase in the steric volume at this position could enhance activity, indicate a beneficial steric effect, and support the consistency of the predicted steric influences. The hydrogen bond donor field map shows blue areas around the amide group and along the linear chain, indicating that the presence of hydrogen bond donor groups in these places is beneficial. In contrast, the yellow areas near the terminal ring suggest that the introduction of such groups in this region would be less favorable for biological activity. In summary, these results emphasize the importance of appropriate distribution of steric volumes and hydrogen bond donor sites in modulating the activity predicted by the CoMSIA models.

### 3.3 2D-QSAR

The 2D-QSAR model was built by using three descriptors SMR, NumHeteroAtoms and FractionCSP3 (Table 5) The model assumes a linear relation and the resulting expression is given in eqn (12). The positive coefficients indicate that these descriptors contribute positively to biological activity. SMR, related to molecular size and polarizability, indicates that increasing the steric bulk will increase activity. Similarly, FractionCSP3, which is representative of molecular three-dimensionality, has a positive contribution, suggesting that more saturated structures can improve the response.

**Table 5:** Molecular descriptors, regression coefficients, and statistical performance of the developed 2D-QSAR model

Variable	Coeff.	Std. Err.	*t*-value	*P* > |*t*|	*Q* ^2^	*R*_test_^2^	*R* ^2^
SMR	0.311	0.132	2.351	0.031			
NumHeteroAtoms	0.165	0.132	1.256	0.226			
FractionCSP3	0.235	0.070	3.358	0.004			
Intercept	6.923	0.065	107.244	0.000			
Values					0.688	0.789	0.788
Mean absolute error					0.336	0.077	—
Root mean squared error					0.407	0.099	—

NumHeteroAtoms is related to the heteroatom content and could be related to hydrogen-bond interactions, although its contribution appears less significant, this descriptor remains relevant for describing specific structural features of the studied compounds.

**Fig. 6:**
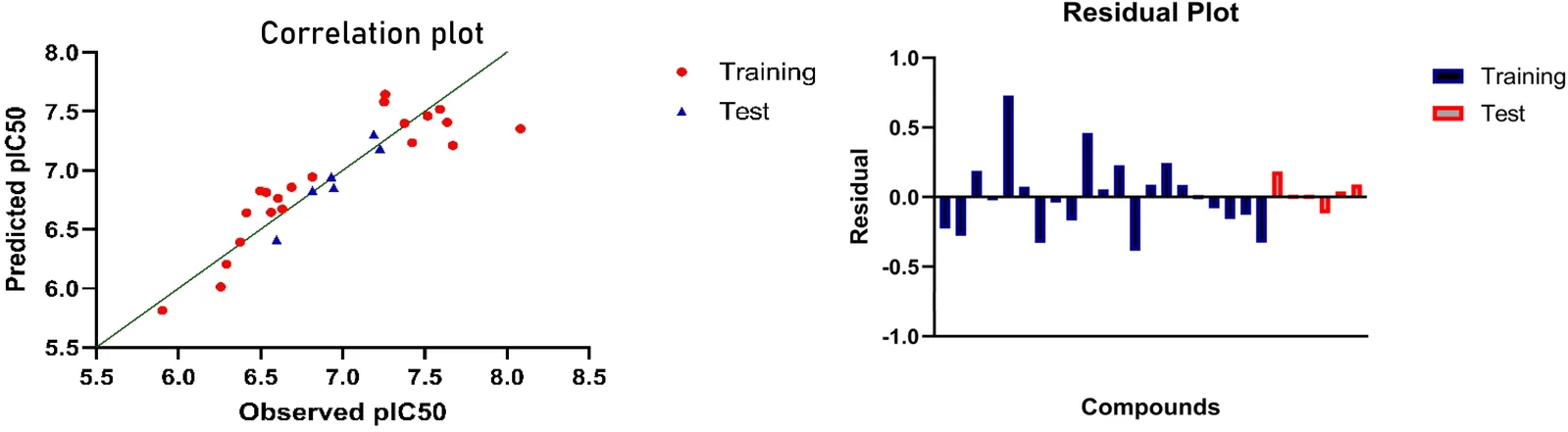
Correlation plot between observed and predicted pIC_50_ values and residuals plot for the training and test sets of the 2D-QSAR model.

Table 5 presents a show of the model's statistical performance. *R*^2^ value indicates a good fit to the training data and *Q*^2^ value confirms the robustness of the model. The *R*_test_^2^ value also shows a good predictive ability on the external set. Fig. 6 shows that good agreement between the experimental and predicted pIC_50_ is obtained for both training and test sets. The residual plot represents evenly scattered about zero with no apparent trend indicating that there is no bias in the predictions. These results are in general agreement with the CoMSIA model and indicate that steric and hydrogen-bond donor effects are the major factors influencing the biological activity.

13 pIC_50_ = 6.923 + 0.311SMR + 0.165NumHeteroAtoms + 0.235FractionCSP3

### 3.4 Randomization analysis

To further substantiate the reliability of the models prior to their use in compound design, Y-randomization tests were performed for the CoMFA, CoMSIA, and 2D-QSAR models to confirm their robustness and non-random nature. The corresponding statistical parameters are summarized in Table 6. The results demonstrate that the *Q*^2^ and *R*^2^ values derived from the randomized models are significantly lower than the original models, suggesting that the observed relationship between molecular descriptors and biological activity is not a consequence of random effects. The mean *Q*^2^ and *R*^2^ values of the randomized models are −0.235 and 0.298 for the CoMFA model, respectively, whereas values of −0.311 and 0.341 were obtained for the CoMSIA model. For the 2D-QSAR model, the mean *Q*^2^ and *R*^2^ values of the randomized models are −0.265 and 0.140, respectively, further supporting the non-random nature of the original model. These distinctions between randomized and original models demonstrate the robustness of established correlations. Moreover, the *cR*_p_^2^ values achieved for CoMFA (0.786), CoMSIA (0.743) and 2D-QSAR (0.719) models are significantly higher than the suggested threshold of 0.5, which confirms the statistical validity and predictive reliability of the developed models. In summary, these findings confirm the validity of both the 3D-QSAR and 2D-QSAR approaches and indicate that the reported structure–activity relationships are based on real chemical trends rather than chance correlations.

**Table 6:** Results of the Y-randomization test for CoMFA, CoMSIA and 2D-QSAR models

Iteration	CoMFA		CoMSIA		2D-QSAR	
	*Q* ^2^	*R* ^2^	*Q* ^2^	*R* ^2^	*Q* ^2^	*R* ^2^
1	−0.315	0.223	−0.485	0.298	0.206	0.134
2	−0.257	0.275	−0.400	0.359	0.041	0.033
3	−0.356	0.288	−0.301	0.269	−0.103	0.221
4	−0.222	0.322	−0.308	0.360	−0.779	0.164
5	−0.027	0.383	−0.061	0.423	−0.690	0.151
Original model	0.665	0.948	0.672	0.932	0.688	0.788
Average values	−0.235	0.298	−0.311	0.341	−0.265	0.141
*cR*_p_^2^	0.786		0.743		0.719	

### 3.5 Bootstrap analysis

**Fig. 7:**
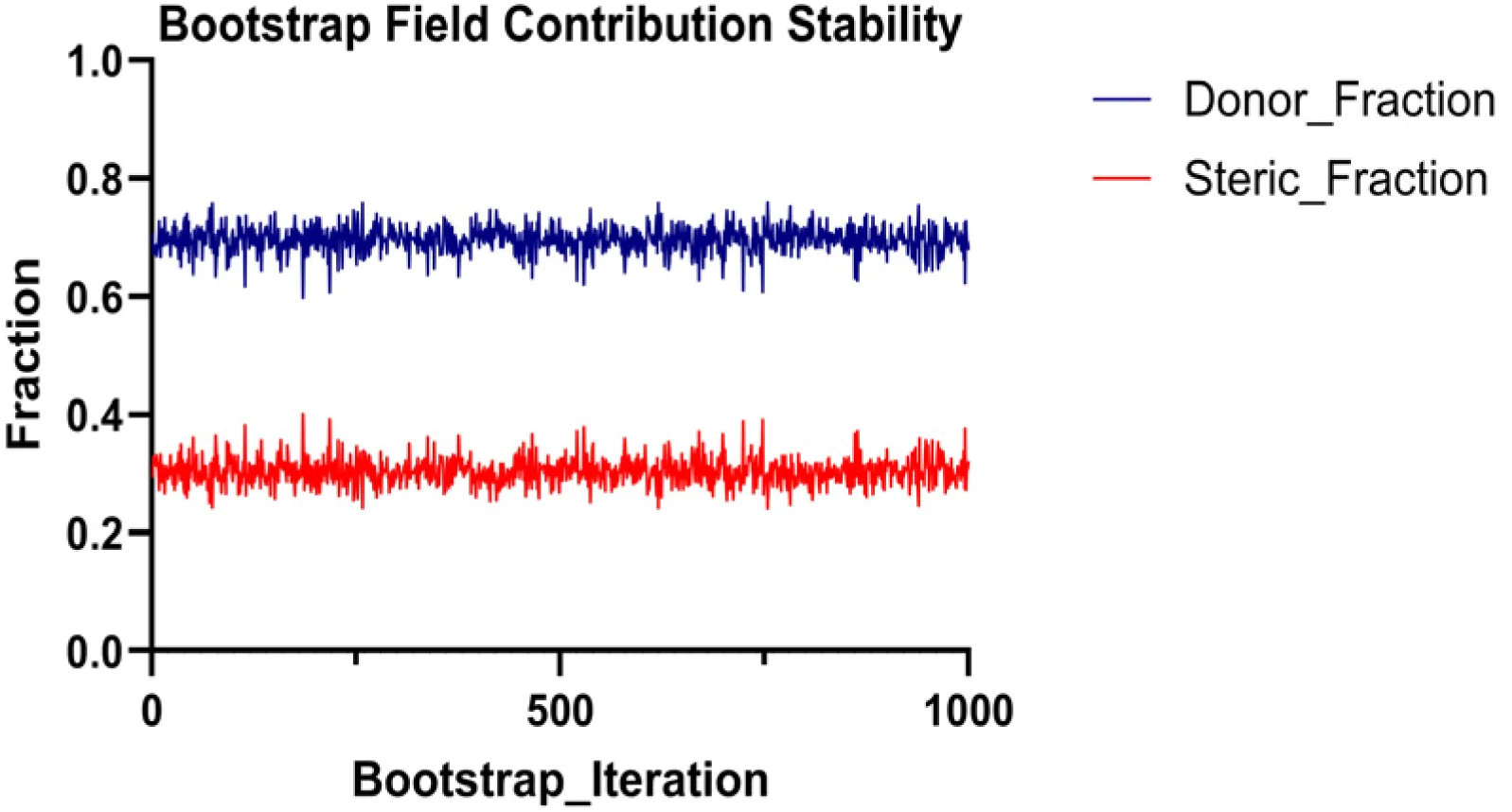
Stability profiles of donor and steric field contributions during bootstrap resampling.

The statistical results of the bootstrap analysis are shown in Table 7. The developed CoMSIA model showed a high mean bootstrap correlation coefficient (*R*^2^ = 0.958) with a low standard deviation (0.023), which indicated the good statistical robustness and reproducibility of the model. In addition, the low average SEE value (0.133) along with its standard deviation (0.101) indicated limited estimation error and acceptable stability during the resampling process. The donor and steric field contributions remained relatively stable during the 1000 bootstrap iterations as shown in Fig. 7. The observed contribution profiles were very consistent with the field contributions obtained for the final CoMSIA model reported in Table 2 where donor and steric fields contributed 0.704 and 0.296 respectively. The small fluctuations observed during the bootstrap procedure are in favor of the stability and the reproducibility of the selected CoMSIA descriptors.

**Table 7:** Bootstrap statistical parameters of the developed CoMSIA model

Parameter	Value
Number of bootstrap runs	1000
Number of components (*N*)	4
Mean bootstrap *R*^2^	0.958
Standard deviation of *R*^2^	0.023
Mean SEE	0.133
Standard deviation of SEE	0.101

### 3.6 Applicability domain analysis

**Fig. 8:**
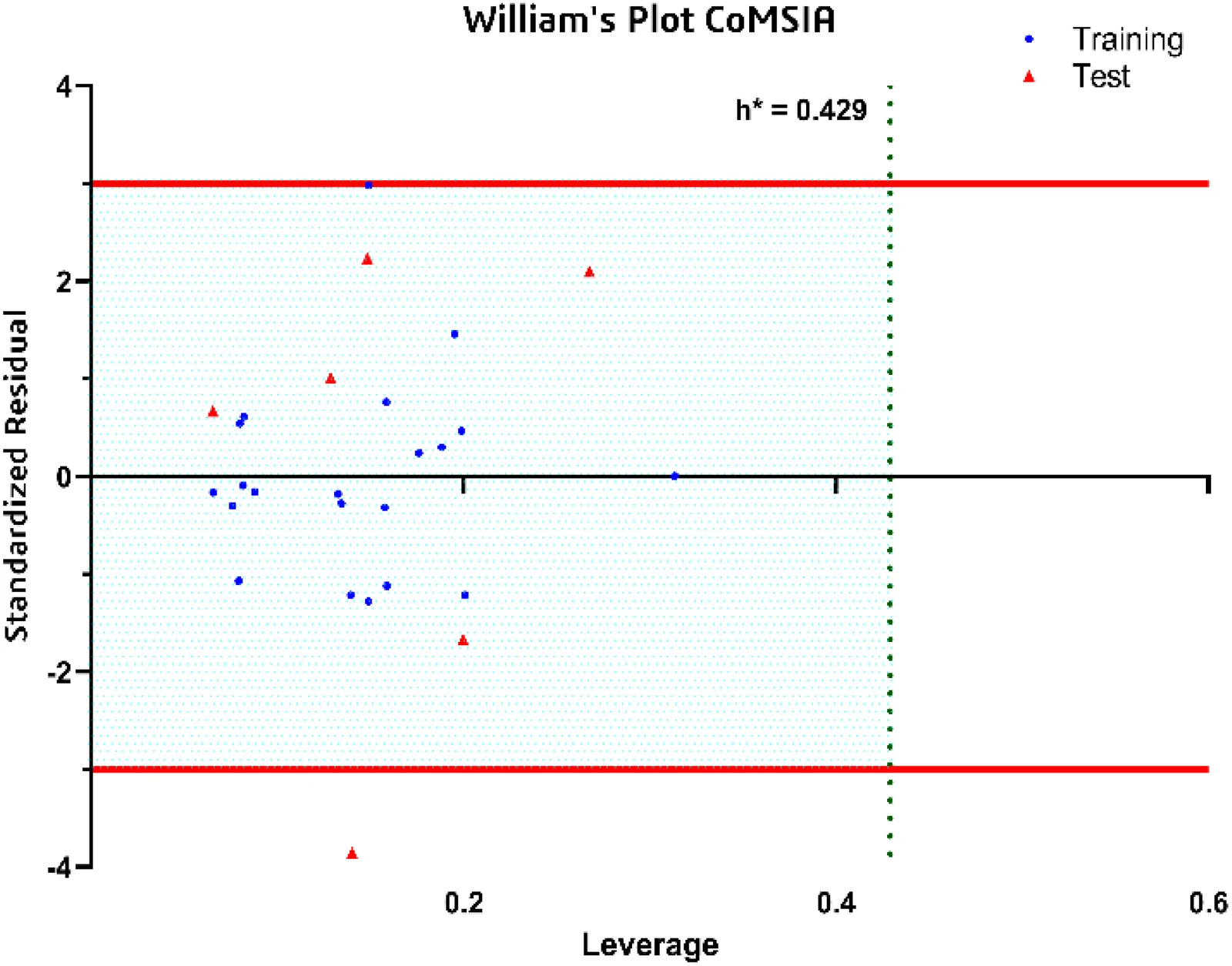
Williams plot of the 3D-QSAR model.

The applicability domain of the constructed 3D-QSAR was assessed using the Williams plot (Fig. 8) which plots the standardized residuals compared to the leverage values. The training set was calculated to have a warning leverage h* of 0.42. The Williams plot indicates that all the training and test compounds are within the critical limit of leverage values indicating that no structurally influential compounds were detected. Most compounds also showed standardized residuals within acceptable limits of ±3. However, one compound from the external test set had a standardized residual slightly below −3 and below the warning leverage threshold. Thus, this compound can be considered as a response outlier rather than a structurally influential compound. However, its position in the applicability domain indicates that the prediction was not extrapolated outside the descriptor space of the training compounds. Overall, these results support robustness and predictability reliability of the developed model.

Although the size of the dataset was relatively small (27 compounds), several validation strategies were applied to guarantee the robustness of the developed models, such as external validation, Y randomization, bootstrap method, applicability domain analysis, and cross-validation. In addition, the consistency between the results obtained from 2D-QSAR and 3D-QSAR gives further support to the reliability of the identified structure–activity relationships. In general, the predictive performance remained acceptable despite the relatively larger residual values observed for some compounds in the external test set, including one response outlier identified in the Williams plot, considering the structural diversity and the limited size of the dataset. The bootstrap analysis further supported the statistical stability and reproducibility of the CoMSIA model and reduced the likelihood of chance correlation or model overfitting. Nevertheless, owing to the limited size and chemically homogeneous nature of the dataset, the predictive ability of the developed models should be considered mainly within the chemical space of structurally related tacrine-carbamate derivatives rather than as a broad generalization to chemically distinct AChE inhibitor classes.

### 3.7 Design of new carbamate derivatives

**Fig. 9:**
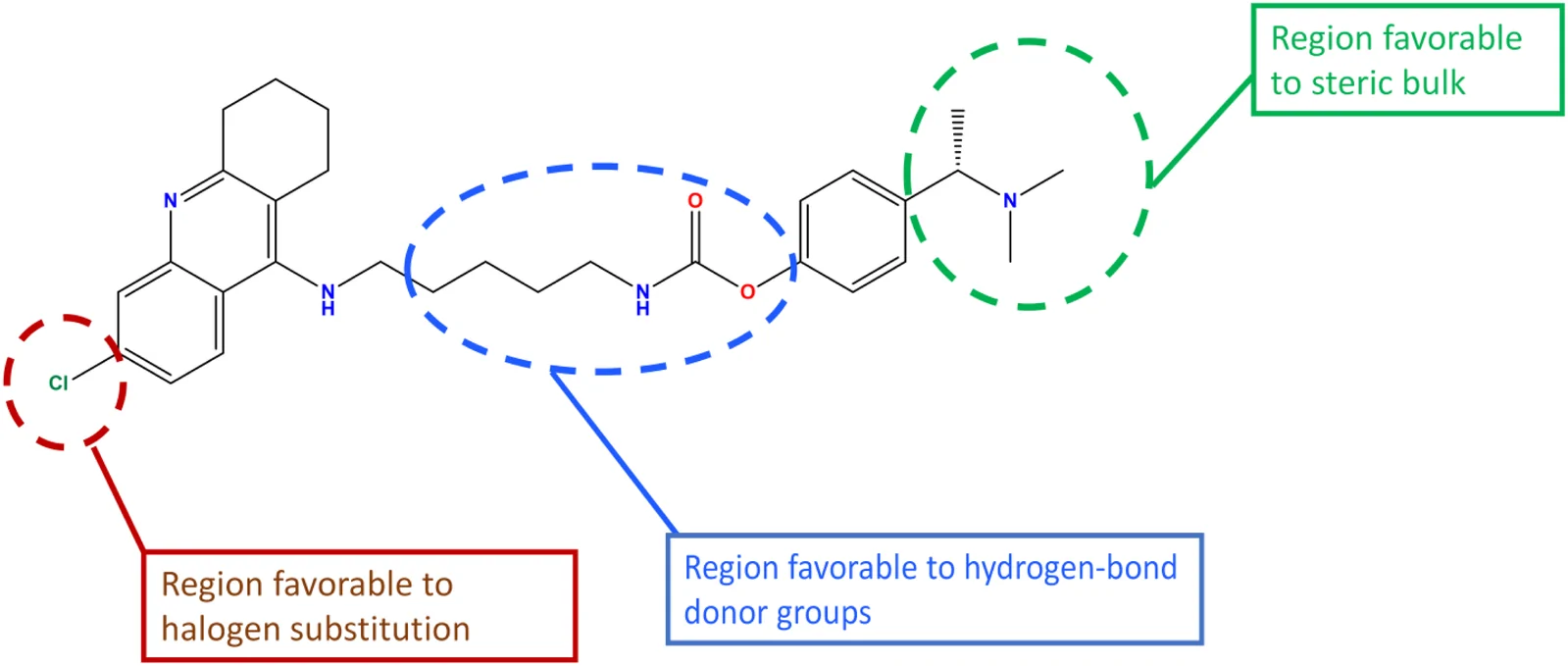
Insights into structure–activity relationships from the 3D-QSAR analysis.

A CoMSIA model was constructed in this study and it was used as a template for designing new carbamate derivatives with improved predicted inhibitory activity against acetylcholinesterase. The structure–activity relationship (SAR) (Fig. 9) emphasized the major role of the steric and hydrogen bond donor contributions, that directly affect the biological response. The analysis yields useful information on chemical features which are favorable for enzymatic inhibition and is useful for rational design of new compounds. From these SAR insights, four new compounds were rationally designed. The newly generated molecules were geometrically optimized and their activities were predicted by the validated CoMSIA model to evaluate their potential inhibitory activity against AChE. In Table 8, the predicted pIC_50_ values of the proposed molecules N1–N4 were slightly higher than that of the reference compound M7 (pIC_50_ = 8.081). Although N1 showed the highest point estimate of the predicted pIC_50_, the differences among N1–N4 ranged from only 0.064 to 0.093 log units, which is lower than the SEE of the CoMSIA model (0.173). Therefore, these compounds should be regarded as having comparable predicted activities within the uncertainty of the model rather than being assigned a definitive QSAR ranking. The predicted values were used as preliminary *in silico* estimates to support the rational design of new candidates and should not be interpreted as definitive evidence of improved biological activity. Although N1–N4 retain the main scaffold of M7, the introduced substituents increase their molecular size and structural complexity. Therefore, their predicted activities require experimental confirmation. The designed compounds were subsequently evaluated using complementary ADMET, DFT, and molecular docking analyses, whereas N2–N4 were further prioritized for molecular dynamics simulations based on the docking results.

**Table 8:** Predicted pIC_50_ values for the newly designed compounds

Compound	Structure	Predicted pIC_50_
M7 (Template)<	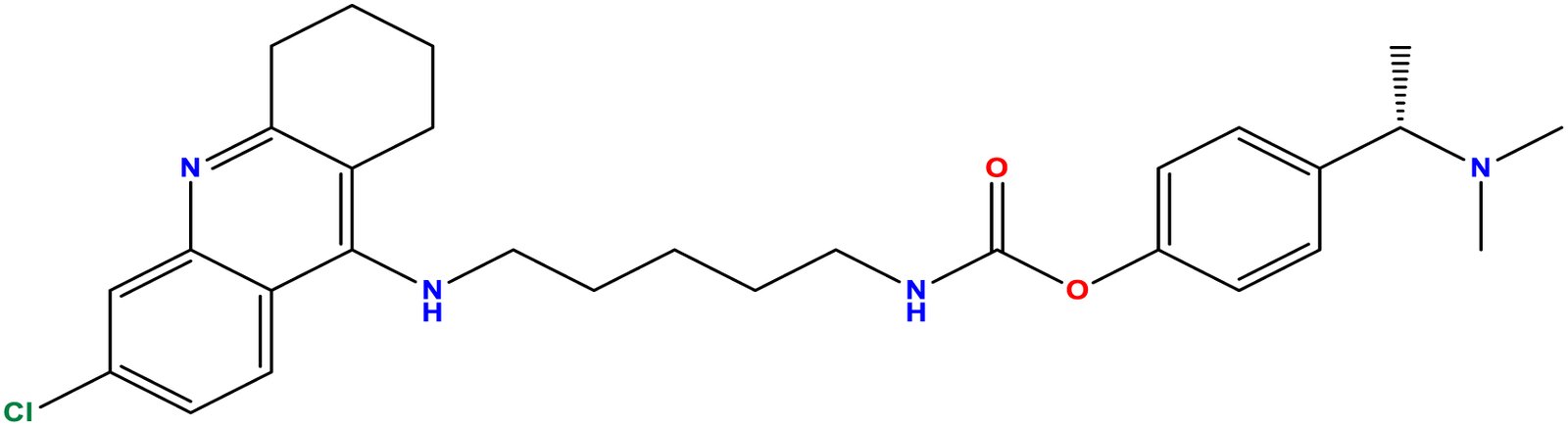	8.081
N1	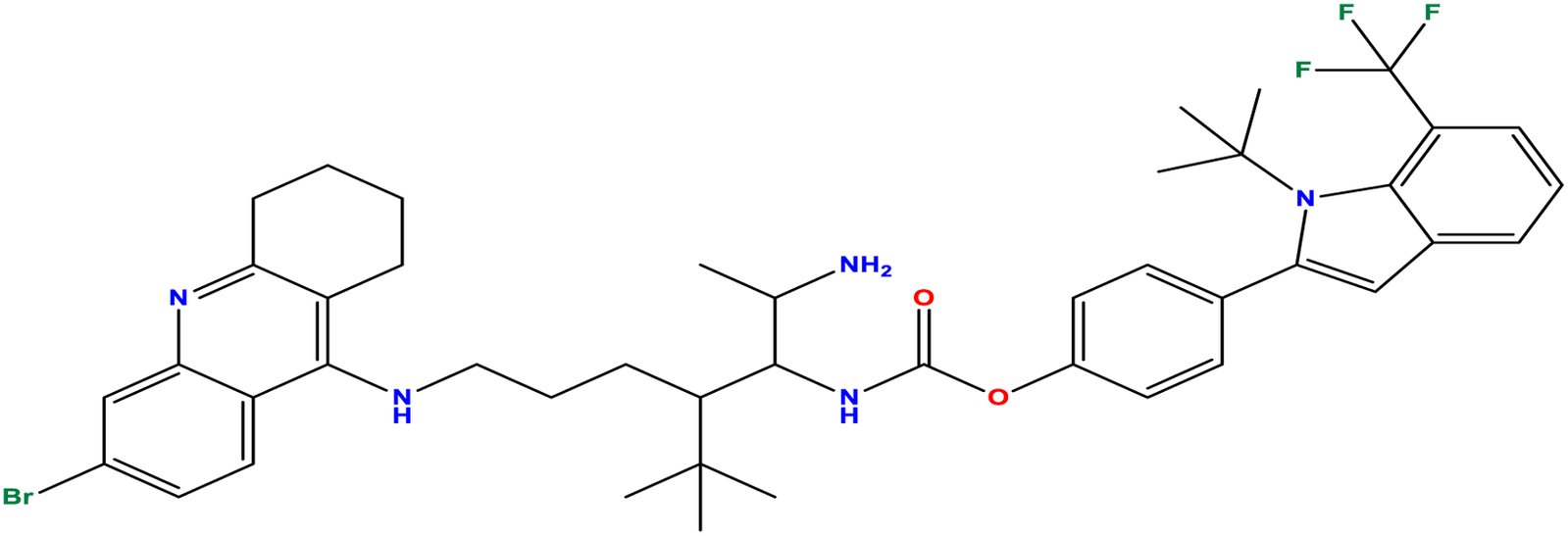	8.204
N2	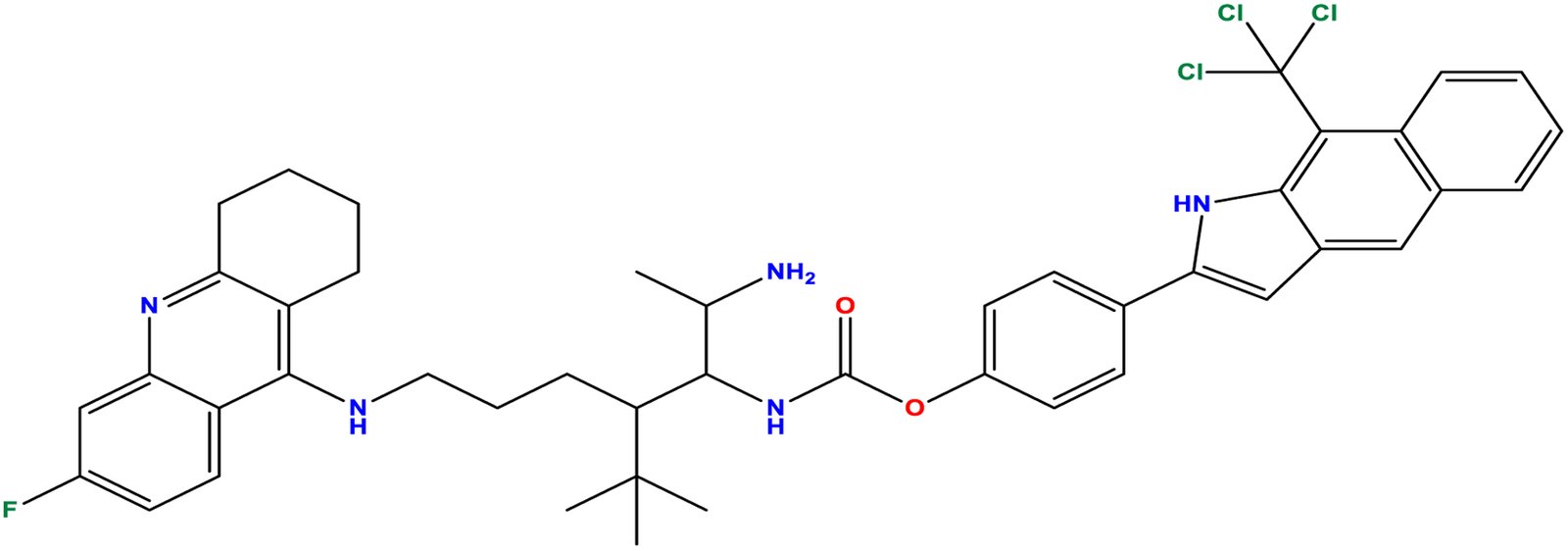	8.140
N3	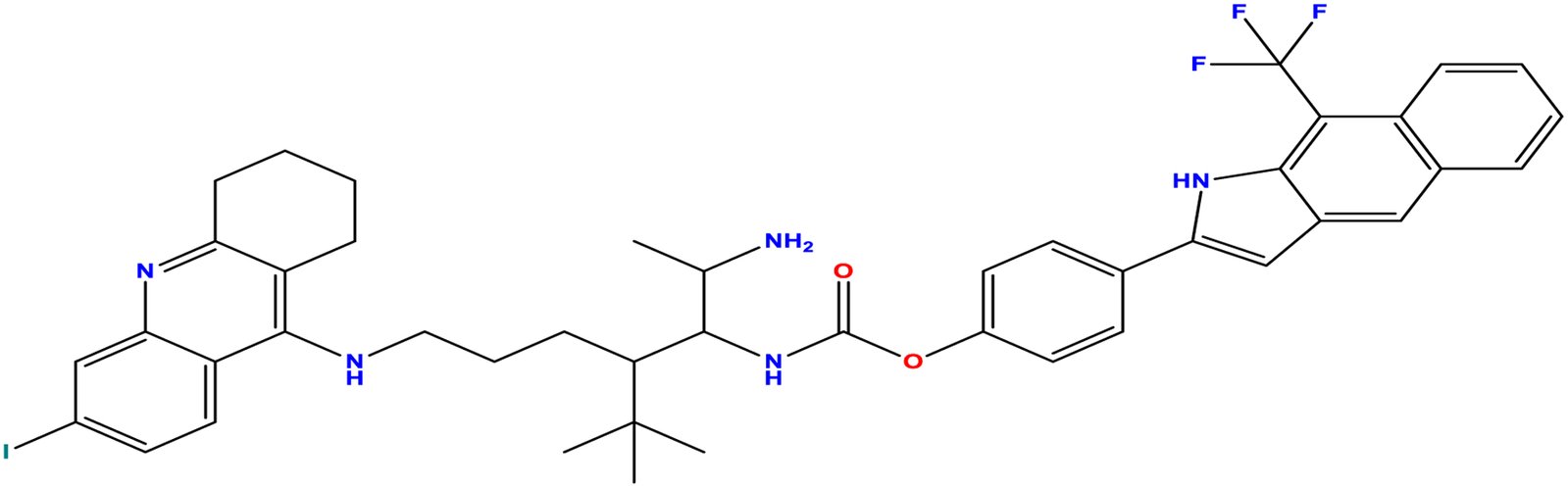	8.121
N4	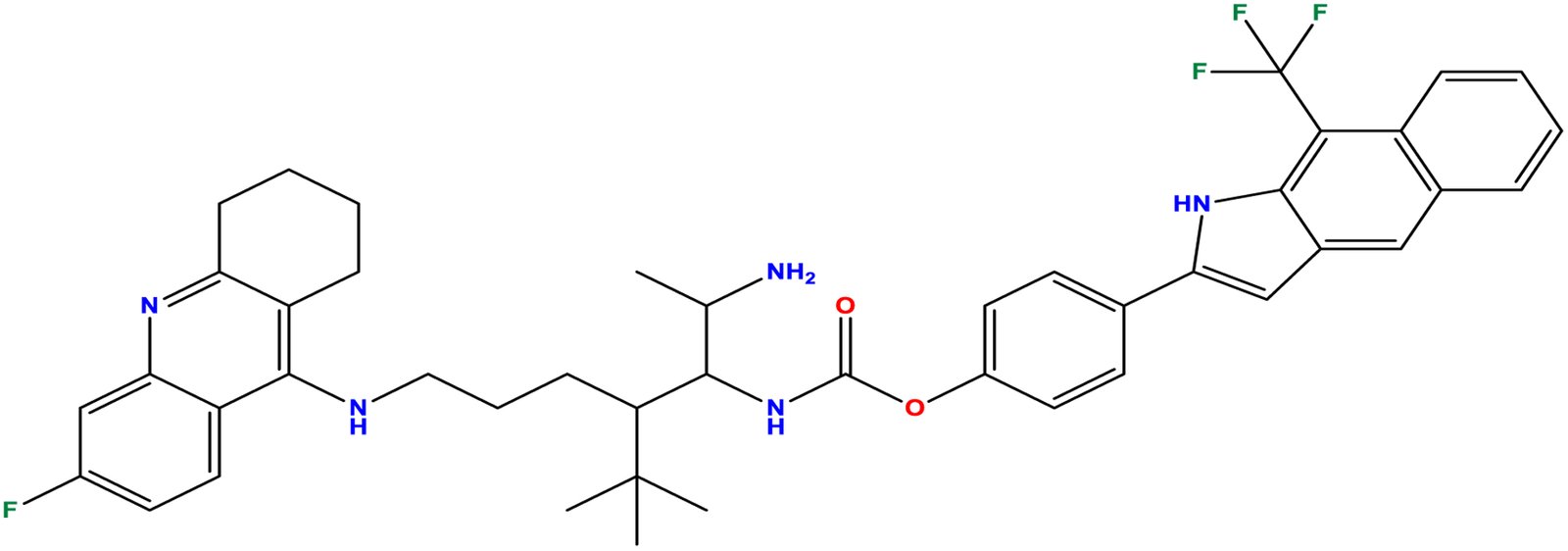	8.111

### 3.8 ADMET predictions

The ADMET properties of the proposed derivatives were studied and compared with the reference compound M7 (Table 9). The new compounds overall show improved aqueous solubility and good intestinal absorption with predicted HIA values up to 100%. The Caco-2 permeability was in the moderate range. Regarding distribution, the predicted log BB values of N1–N4 (−1.305 to −1.540) were markedly lower than that of M7 (−0.269), corresponding to approximately 11-19-fold lower predicted brain-to-blood concentration ratios. This reduced predicted BBB permeability represents a major limitation for their potential development as CNS-targeted candidates. All compounds were predicted to be substrates for CYP3A4, indicating metabolism *via* cytochrome P450 enzymes.

**Table 9:** Results of ADMET predictions for the newly designed derivatives

ADMET Parameters	Compounds				
	M7	N1	N2	N3	N4
**Absorption properties**					
Aqueous solubility (log mol L^−1^)	−4.874	−3.35	−2.948	−2.973	−2.969
Caco-2 permeability (log *P* app)	0.549	0.453	0.341	0.354	0.504
Human intestinal absorption (%)	86.127	100	100	100	100

**Distribution profile**					
Blood brain barrier permeability (log BB)	−0.269	−1.305	−1.54	−1.401	−1.432

**Metabolic behavior**					
CYP 2D6 (substrate) (Yes or no)	YES	NO	YES	YES	YES
CYP 3A4 (substrate) (Yes or no)	YES	YES	YES	YES	YES
CYP 1A2 (inhibitor) (Yes or no)	NO	NO	YES	NO	YES
CYP 2C19 (inhibitor) (Yes or no)	YES	YES	YES	YES	YES
CYP 2C9 (inhibitor) (Yes or no)	NO	NO	NO	NO	NO

**Elimination profile**					
Predicted total clearance (log ml min^−1^ kg^−1^)	1.169	0.106	0.178	0.281	0.248

**Toxicological assessment**					
Mutagenicity (AMES test) (Yes or no)	NO	NO	NO	NO	NO
hERG inhibition (Yes/No (Yes or no)	NO	NO	NO	NO	NO
Max tolerated dose (log mg kg^−1^ day^−1^)	−0.164	0.296	0.395	0.379	0.372
Skin sensitisation (Yes or no)	No	No	No	No	No

**Synthetic feasibility and MCE-18**					
Synthetic accessibility (easy/Hard)	Easy	Easy	Easy	Easy	Easy
MCE-18	73.381	137.5	141.0	141.0	141.0

The designed derivatives also showed lower clearance values indicating a longer residence time. Toxicity predictions showed no risk for mutagenicity, hERG inhibition and skin sensitization. Additionally, the higher maximum tolerated doses and acceptable synthetic accessibility scores indicate a good safety profile and reasonable synthetic feasibility of the proposed compounds. The MCE-18 values suggest an increase in the molecular complexity with respect to the reference compound, indicating an increased structural diversity of the designed derivatives.

Overall, the N1–N4 derivatives showed favorable predictions for selected ADMET parameters, particularly aqueous solubility, intestinal absorption, clearance, and toxicity endpoints. However, their markedly reduced predicted BBB permeability prevents them from being considered to have improved overall pharmacokinetic profiles and represents a major limitation for CNS-targeted development. These results should be considered preliminary computational predictions requiring further structural optimization and experimental validation.

### 3.9 DFT results

The electronic properties of compounds N1–N4 were studied by density functional theory (DFT) calculations with M7 as the reference compound. Fig. 11 displays the HOMO, LUMO, and electrostatic potential (ESP) maps.

The frontier orbitals of M7 are largely localized on the aromatic ring, which implies the dominant role of the aromatic ring in the electronic distribution. The orbitals are located over the carbamate linker and terminal ring system, similar to the localization pattern for N2–N4. N1 shows a more distinct spatial separation between the HOMO and LUMO, indicating a different electronic distribution from the other derivatives. In the ESP maps, the electron-rich regions are mainly over the aromatic ring and carbamate group. The electron-deficient regions are more pronounced near the amine group in the designed compounds.

**Fig. 10:**
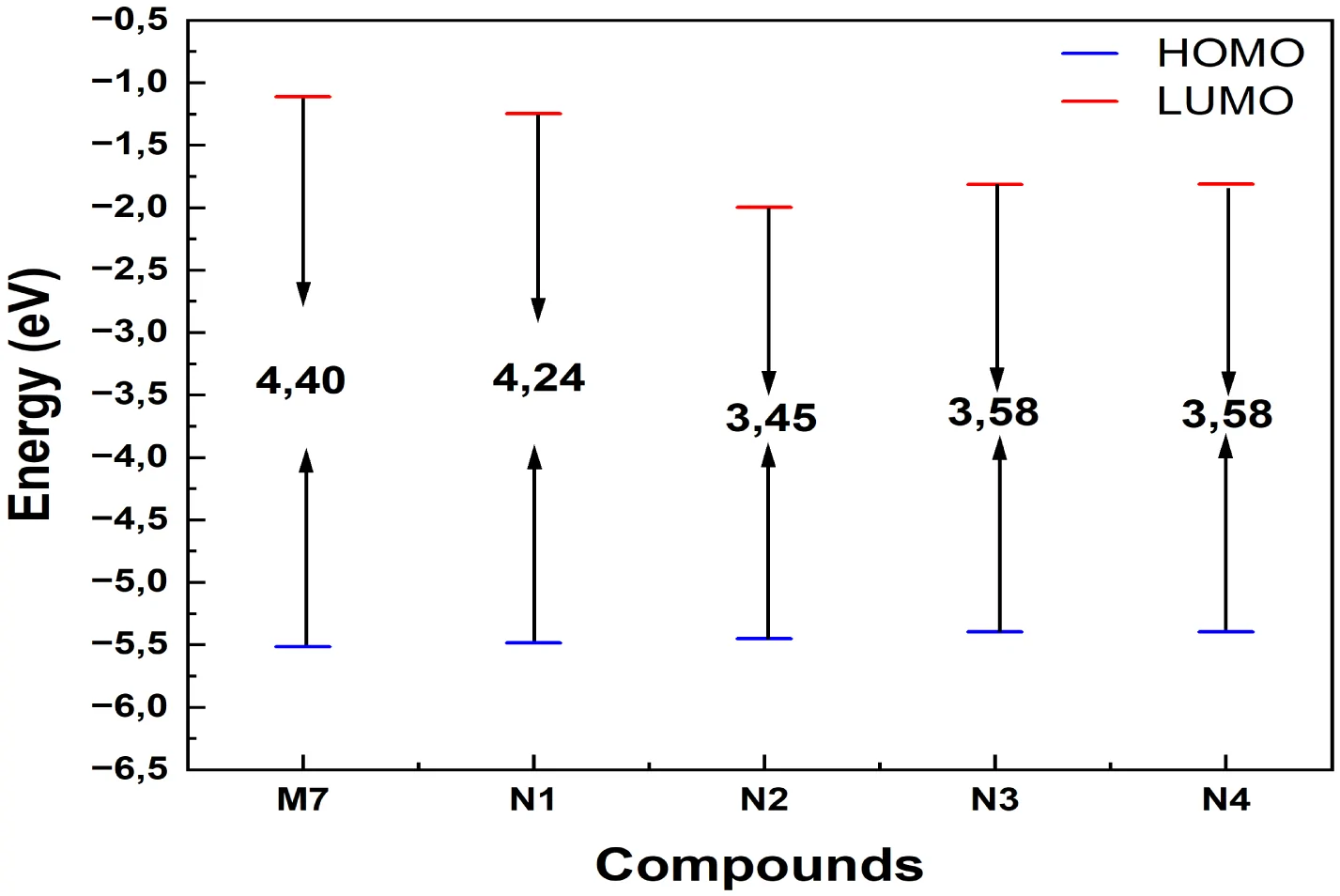
Comparison of HOMO–LUMO energy gaps for the reference compound M7 and the predicted derivatives.

The calculated energy parameters (Table 10) show that all derivatives have lower gas-phase HOMO–LUMO gaps than M7 (4.403 eV). Specifically, N2–N4 have gap values in the range of 3.45–3.59 eV and show higher calculated softness and electrophilicity. As illustrated in Fig. 10, these results reflect differences in the electronic characteristics of the isolated molecules. However, they do not establish a relationship with AChE inhibitory activity.

**Table 10:** Electronic descriptors calculated using density functional theory (DFT)

	M7	N1	N2	N3	N4
HOMO (eV)	−5.516	−5.487	−5.452	−5.396	−5.397
LUMO (eV)	−1.113	−1.249	−2.000	−1.815	−1.813
Band gap (eV)	4.403	4.238	3.453	3.581	3.585
Ionization energy (eV)	5.516	5.487	5.452	5.396	5.397
Electron affinity (eV)	1.113	1.249	2.000	1.815	1.813
Global hardness (eV)	2.202	2.119	1.726	1.791	1.792
Global softness (eV^−1^)	0.227	0.236	0.290	0.279	0.279
Chemical potential (eV)	−3.314	−3.368	−3.726	−3.605	−3.605
Electronegativity (eV)	3.314	3.368	3.726	3.605	3.605
Global electrophilicity index (eV)	2.495	2.676	4.021	3.630	3.626

**Fig. 11:**
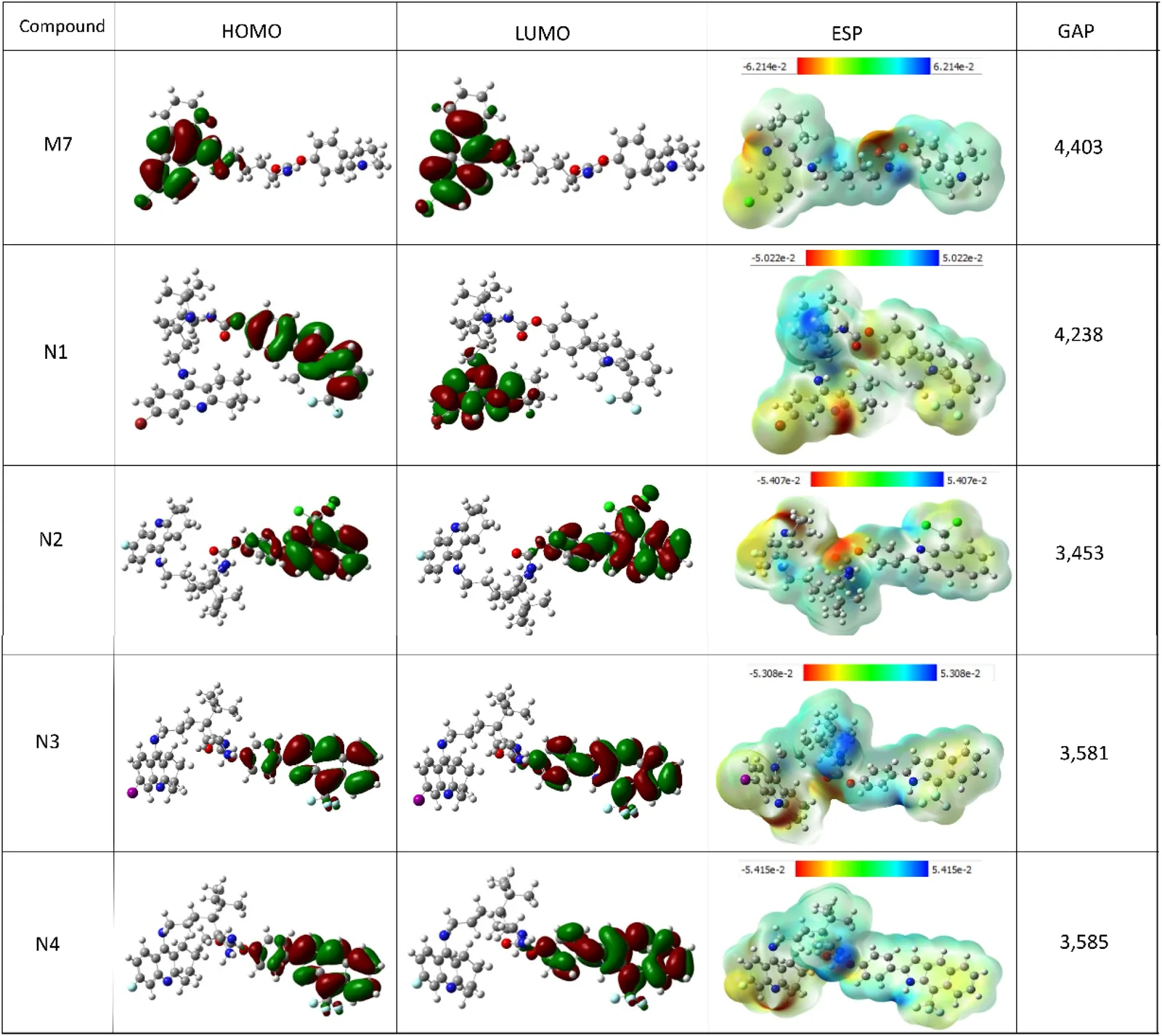
Frontier molecular orbitals (HOMO–LUMO) and electrostatic potential (ESP) maps of the studied compounds.

The distribution of the frontier orbitals highlights the main electronic regions of the studied compounds and allows comparison of their electronic organization. These electronic descriptors provide complementary information on the electronic behavior of the designed compounds. However, a lower HOMO–LUMO gap does not directly indicate stronger binding affinity or higher inhibitory activity, because ligand binding also depends on steric complementarity, intermolecular interactions, conformational dynamics, and solvation effects.

### 3.10 Molecular redocking analysis

**Fig. 12:**
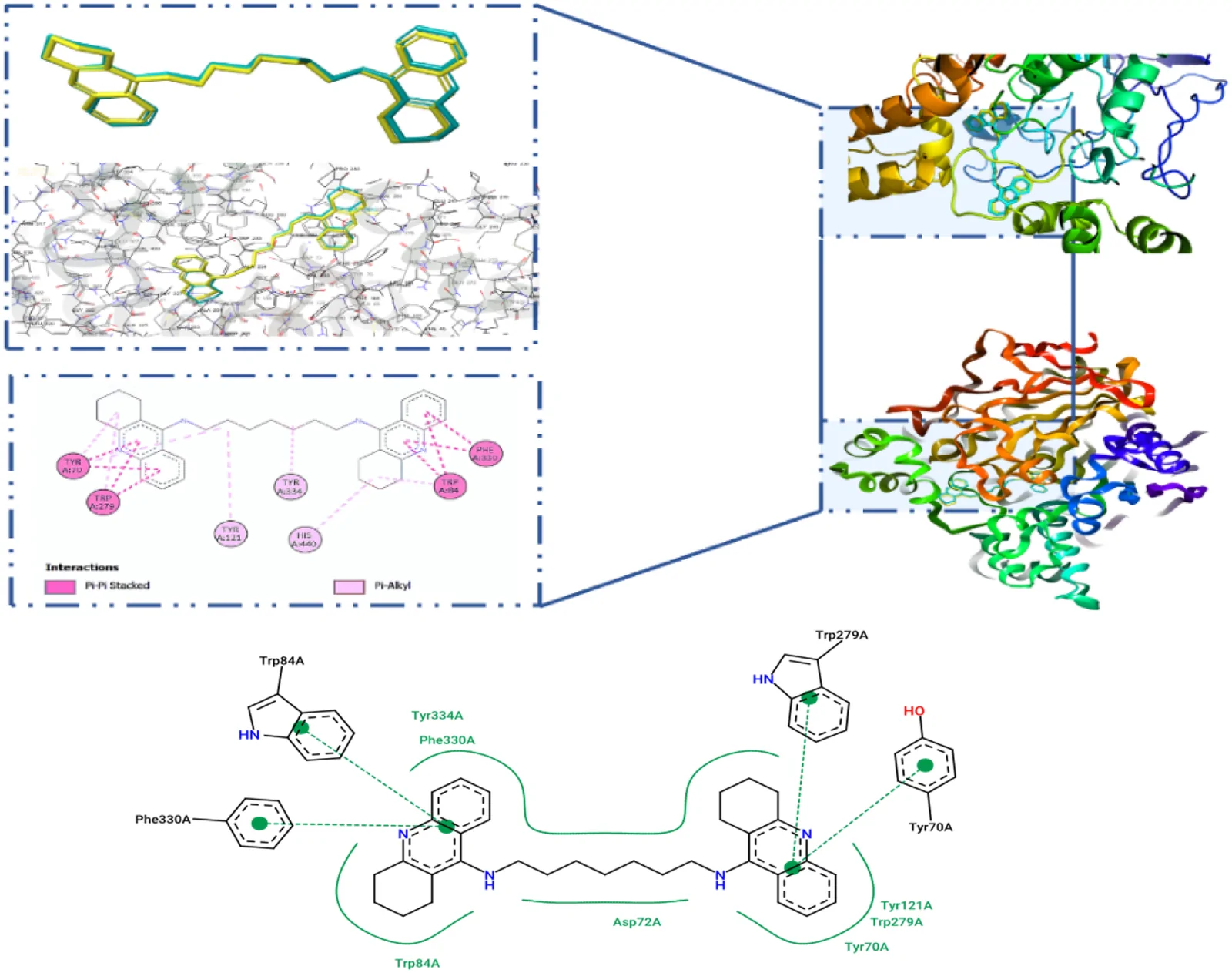
Superimposition of the redocked ligand (cyan) and the crystallographic ligand (yellow) within the active site of *Torpedo californica* acetylcholinesterase (TcAChE; PDB ID: 2CKM), together with the corresponding 2D interaction diagram of the crystallographic ligand.

**Fig. 13:**
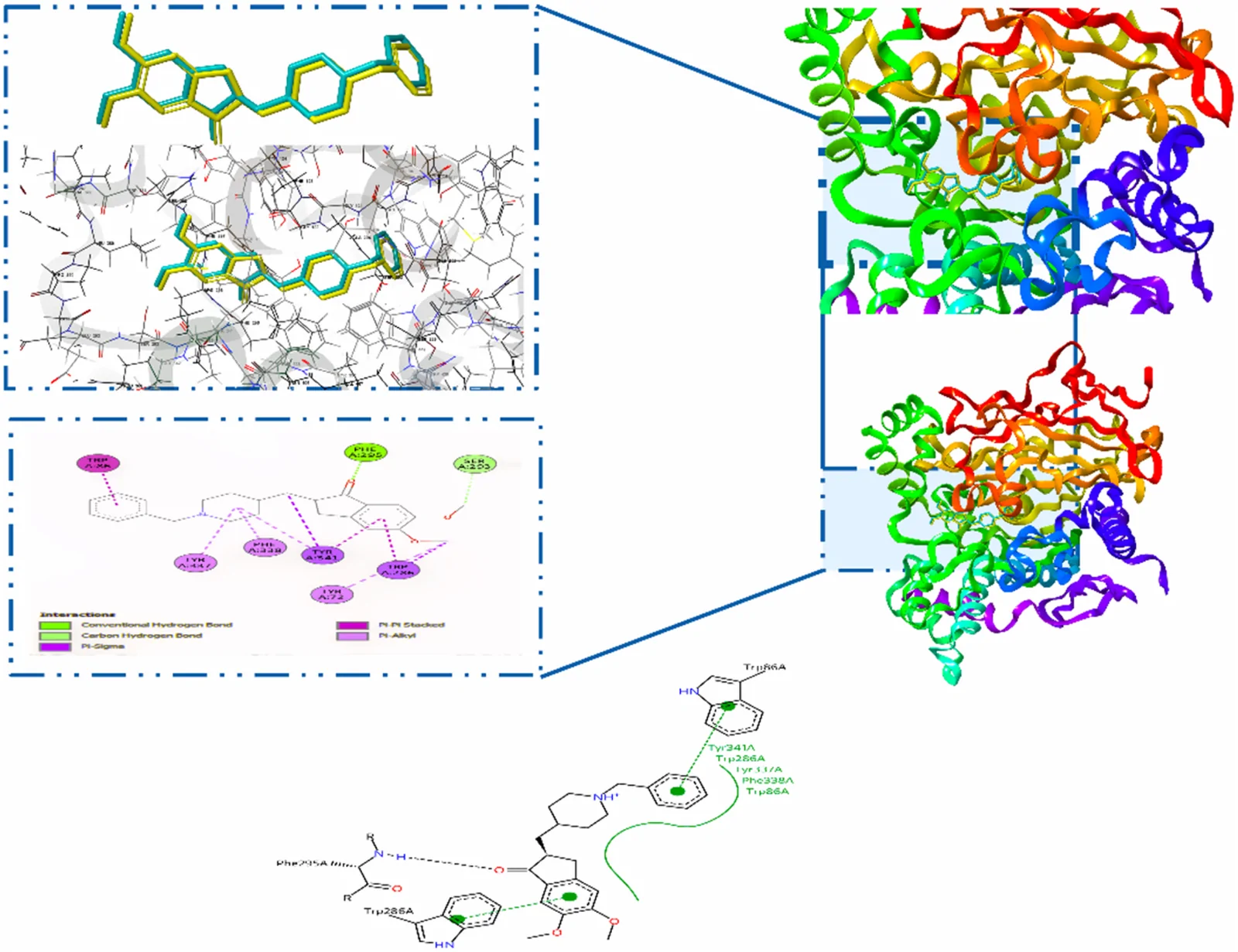
Superimposition of the redocked donepezil ligand (cyan) and the crystallographic donepezil ligand (yellow) within the active site of human acetylcholinesterase (hAChE; PDB ID: 4EY7), together with the corresponding 2D interaction diagram of the crystallographic ligand.

The docking protocol for *Torpedo californica* acetylcholinesterase (TcAChE, PDB ID: 2CKM) was initially validated by redocking its co-crystallized ligand. The ligand AA7 was removed and redocked into the active site using AutoDock4. The root mean square deviation (RMSD) between the redocked and crystallographic poses was 0.404 Å, indicating excellent conformational superposition between the two poses. This value, well below the commonly accepted threshold of 2 Å, confirms the accuracy and validity of the docking protocol. The docking protocol for human acetylcholinesterase (hAChE, PDB ID: 4EY7) was additionally validated by removing and redocking the co-crystallized donepezil ligand into the active site using the same docking procedure. The RMSD between the crystallographic and redocked donepezil poses was 0.393 Å, confirming the ability of the protocol to reproduce the experimentally observed binding mode within the human AChE active site. Fig. 12 illustrates the superposition of the redocked AA7 ligand (cyan) onto its crystallographic pose (yellow) within the TcAChE active site. Similarly, Fig. 13 shows the superposition of the redocked donepezil pose onto its crystallographic orientation within the hAChE active site. Furthermore, the 2D interaction analysis of the redocked AA7-TcAChE complex revealed π–π stacking and π-alkyl interactions involving Tyr70, Trp279, Tyr121, Phe330, Tyr334, Trp84, and His440. For the hAChE system, the 2D interaction analysis of the redocked donepezil complex revealed a conventional hydrogen bond with Phe295 and a carbon–hydrogen bond with Ser293. The ligand also formed π–π stacking interactions with Trp86 and Trp286, π-alkyl interactions with Tyr72, Tyr337, and Phe338, as well as π-sigma interactions involving Tyr341 and Trp286. Collectively, these findings confirm the reliability of the redocking protocols for reproducing the crystallographic binding modes in both TcAChE and hAChE and support their use for predicting the binding modes of the newly designed carbamate derivatives.

### 3.11 Molecular docking results

**Fig. 14:**
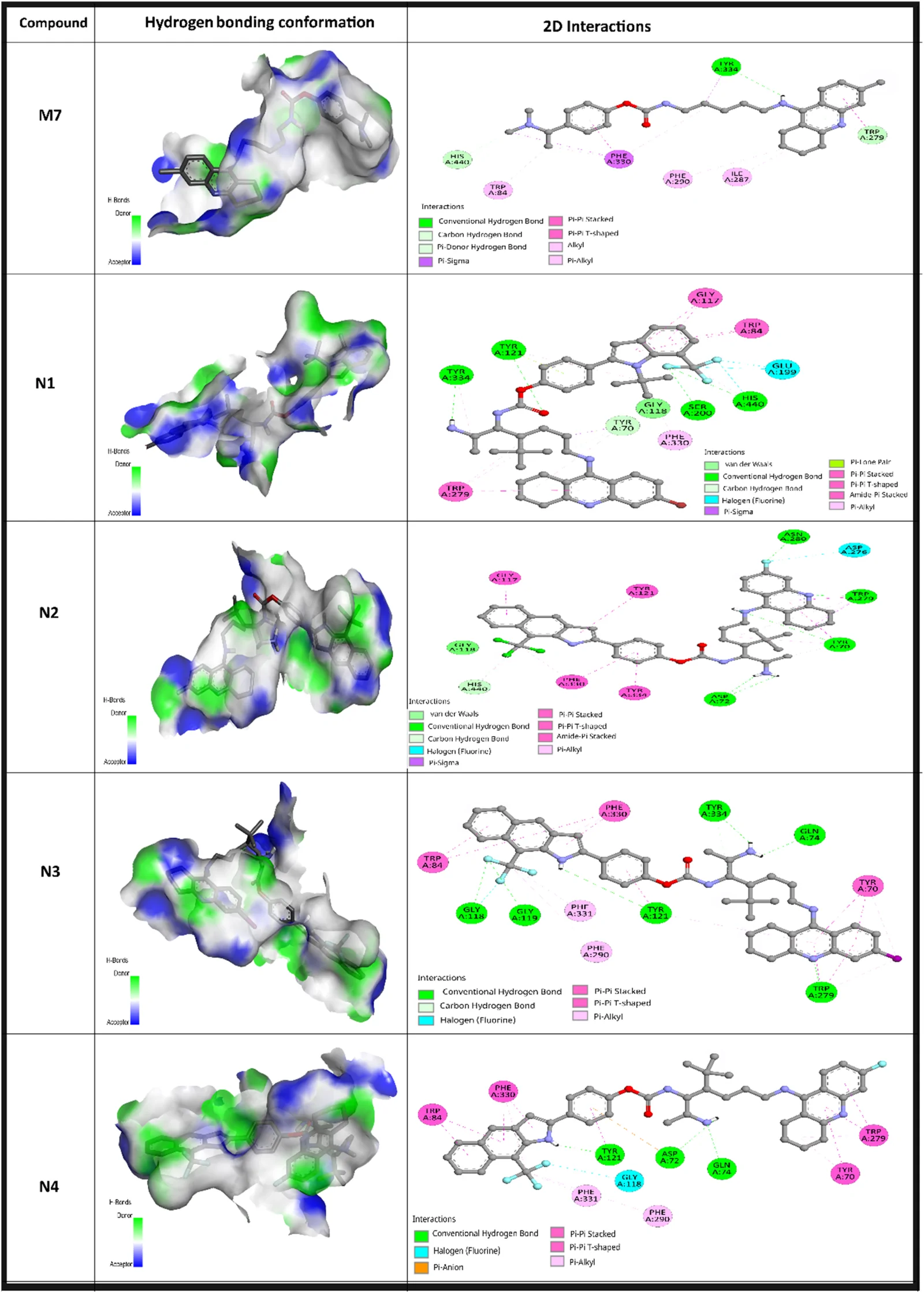
Two- and three-dimensional representations from molecular docking showing ligand-2CKM interactions.

**Fig. 15:**
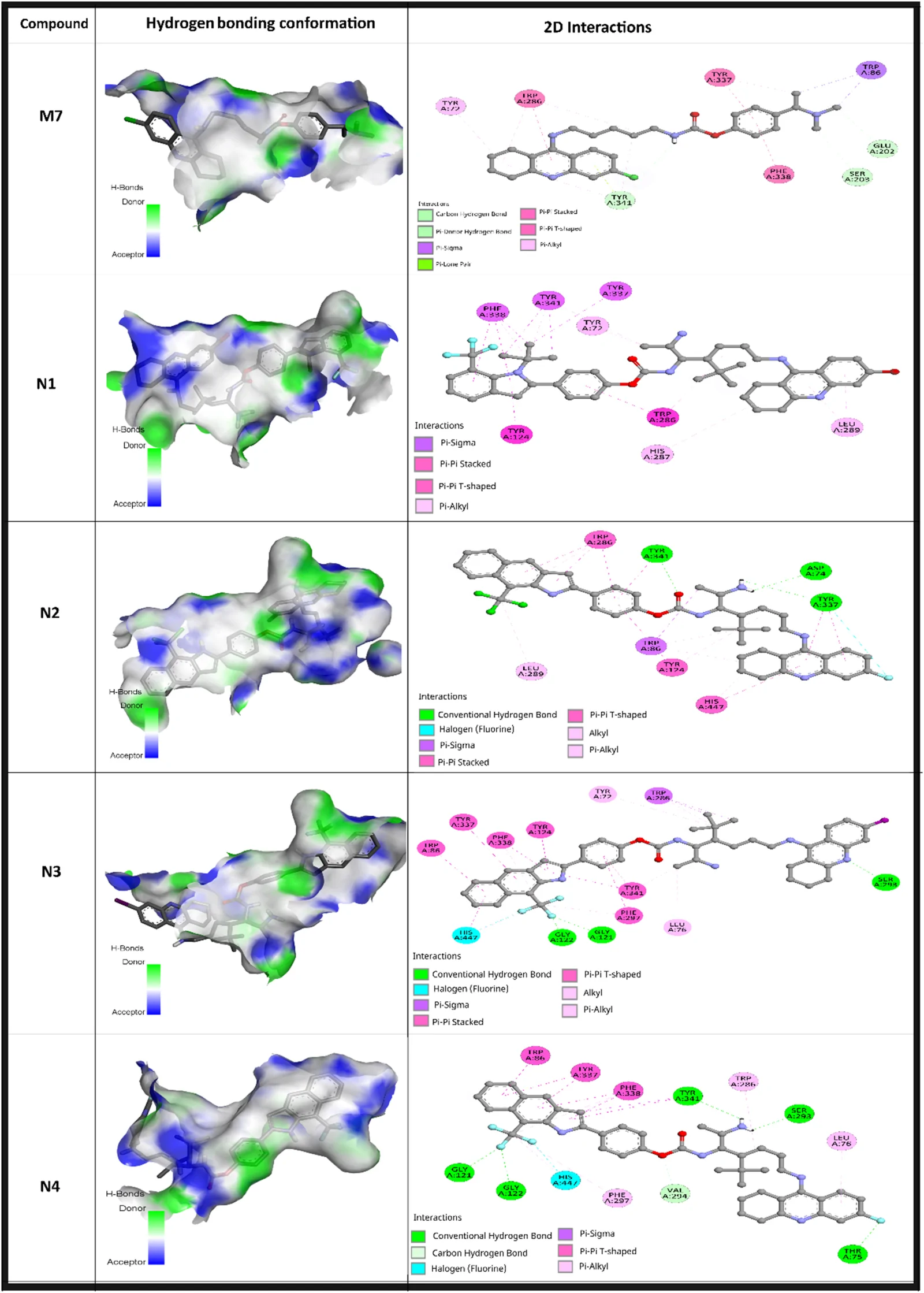
Two- and three-dimensional representations from molecular docking showing ligand-4EY7 interactions.

Docking analysis indicated that all ligands were accommodated within the active-site gorge of both Torpedo californica acetylcholinesterase (TcAChE; PDB ID: 2CKM) and human acetylcholinesterase (hAChE; PDB ID: 4EY7). The corresponding binding energies and interacting residues are summarized in Table 11 and 12, whereas the 2D interaction profiles and 3D surface representations of the ligand-2CKM and ligand-4EY7 complexes are presented in Fig. 14 and 15, respectively.

**Table 11:** Binding energies and key interacting residues of ligand-2CKM complexes obtained from molecular docking

Compound	M7	N1	N2	N3	N4
Binding energy (kcal mol^−1^)	−9.20	−9.58	−11.26	−10.01	−11.15
Hydrogen bond interaction	Tyr334	Tyr334, Tyr121, His440, Ser200	Asp72, Tyr70, Trp279, Asn280	Gly118, Gly119, Tyr121, Tyr334, Gln74, Trp279	Asp72, Gln74, Tyr121
Hydrophobic interactions (alkyl/π-alkyl)	Phe290, Ile287, Trp84, Phe330	Tyr70, Trp279, Phe330, Tyr121, His440	Phe330, His440, Tyr70, Trp279	Phe331, Phe290, Tyr70, Trp279, Tyr121, Phe330	Phe290, Phe331, Tyr70, Trp279, Phe330
π-Sigma	Phe330, Tyr334	Tyr70	Trp279	—	—
π–π stacked, π–π T-shaped and amide π stacked	Trp279, Phe330	Trp279, Trp84, Gly117	Phe330, Tyr334, Tyr121, Gly117, Tyr70, Trp279	Trp84, Phe330, Tyr70, Trp279	Tyr70, Trp279, Phe330, Trp84, Tyr121
Miscellaneous interactions	His440, Trp279	Glu199 (halogen), Ser200 (halogen), Tyr70, Tyr121	Asp276 (halogen), His440	Gly118 (halogen), Tyr334	Gly118, (halogen), Asp72 (Pi-anion)

**Table 12:** Binding energies and key interacting residues of ligand-4EY7 complexes obtained from molecular docking

Compound	M7	N1	N2	N3	N4
Binding energy (kcal mol^−1^)	−9.76	−10.44	−10.69	−10.99	−11.99
Hydrogen bond interaction	—	—	Asp74, Tyr337, Tyr341	Gly121, Gly122, Ser293	Gly121, Gly122, Tyr341, Ser293, Thr75
Hydrophobic interactions (alkyl/π-alkyl)	Tyr72, Trp286, Tyr337, Tyr341	Phe338, Tyr341, Tyr72, Trp286, His287, Leu289	Trp286, Trp86, Tyr124, Leu289, His447	Tyr72, Trp286, Phe338, Tyr341, Phe297, Leu76	Trp286, His447, Phe297, Leu76, Phe338
π-Sigma	Trp86	Phe338, Tyr341, Tyr337	Trp86	Trp286	Tyr341
π–π stacked and π–π T-shaped	Trp286, Tyr337, Phe338	Phe338, Trp286, Tyr124	Trp286, Tyr341, Tyr337, Tyr124, His447	Trp86, Tyr337, Phe338, Tyr124, Tyr341, Phe297, His447	Trp86, Tyr337, Phe338, Tyr341
Miscellaneous interactions	Tyr341 (pi-donor H-bond, pi–lone pair), Glu202, Ser203 (C–H bonds)	—	Tyr337 (halogen)	His447 (halogen)	His447 (halogen), Val294 (C–H bond)

For TcAChE, the predicted binding energies ranged from −9.20 to −11.26 kcal mol^−1^. The N1-2CKM, N2-2CKM, N3-2CKM, and N4-2CKM complexes exhibited more favorable predicted docking energies than the M7-2CKM complex, with N2-2CKM showing the lowest value of −11.26 kcal mol^−1^. These docking energies represent relative computational estimates and should not be interpreted as direct experimental measurements of binding affinity.

The M7-2CKM complex formed a hydrogen bond with Tyr334, together with hydrophobic and aromatic contacts involving Trp84, Phe330, and Trp279. The designed derivatives displayed a broader range of non-covalent contacts than the M7-2CKM complex. N1-2CKM exhibited a predicted docking energy of −9.58 kcal mol^−1^ and combined hydrogen-bonding, aromatic, and hydrophobic contacts. N2-2CKM formed several hydrogen bonds together with hydrophobic and aromatic contacts involving key TcAChE residues. N3-2CKM formed multiple hydrogen bonds together with hydrophobic and aromatic contacts, consistent with a well-defined predicted binding orientation. N4-2CKM also formed several hydrogen-bonding, hydrophobic, and aromatic interactions, consistent with its predicted binding orientation.

Docking against hAChE yielded predicted binding energies ranging from −9.76 to −11.99 kcal mol^−1^, as summarized in Table 12. All designed derivatives exhibited more favorable docking energies than the M7-4EY7 complex, with N4-4EY7 showing the lowest predicted value of −11.99 kcal mol^−1^.

The M7-4EY7 and N1-4EY7 complexes were mainly characterized by hydrophobic and aromatic contacts, with representative interactions involving Trp286, Phe338, Tyr337, Tyr341, and Leu289 across the two complexes; neither complex formed conventional hydrogen bonds. The M7-4EY7 complex additionally displayed a π-donor hydrogen bond and a π-lone-pair interaction with Tyr341, together with carbon–hydrogen bonds involving Glu202 and Ser203. In contrast to M7-4EY7 and N1-4EY7, the N2-4EY7, N3-4EY7, and N4-4EY7 complexes established conventional hydrogen bonds as well as hydrophobic and aromatic interactions. N2-4EY7 formed hydrogen bonds with Asp74, Tyr337, and Tyr341, whereas N3-4EY7 and N4-4EY7 shared hydrogen-bonding contacts with Gly121, Gly122, and Ser293. These complexes also exhibited aromatic interactions involving representative residues such as Trp286, Phe338, Tyr337, and Tyr341. Furthermore, halogen interactions with His447 were noted in the N3-4EY7 and N4-4EY7 complexes.

Overall, all designed derivatives exhibited more favorable predicted docking energies than the corresponding M7 complex for each receptor structure. However, the lowest-energy ligand depended on the protein model, with N2-2CKM showing the most favorable value for TcAChE and N4-4EY7 showing the most favorable value for hAChE, indicating differences in ligand recognition between the two AChE structures.

The docking analysis revealed π-mediated contacts and hydrogen-bonding interactions within the binding sites. Although N1 showed the highest point estimate of the QSAR-predicted pIC_50_, the differences among N1–N4 were smaller than the SEE of the CoMSIA model, indicating comparable predicted activities within the uncertainty of the model. Furthermore, N1 exhibited less favorable docking energies than N2–N4 against both receptor structures. The selection of compounds for molecular dynamics simulations was therefore based on the combined assessment of docking energies and interaction profiles rather than on the QSAR ranking alone. Accordingly, M7 and N2–N4 were selected for the subsequent MD simulations with both TcAChE and hAChE, whereas N1 was not included.

### 3.12 Molecular dynamics results

**Fig. 16:**
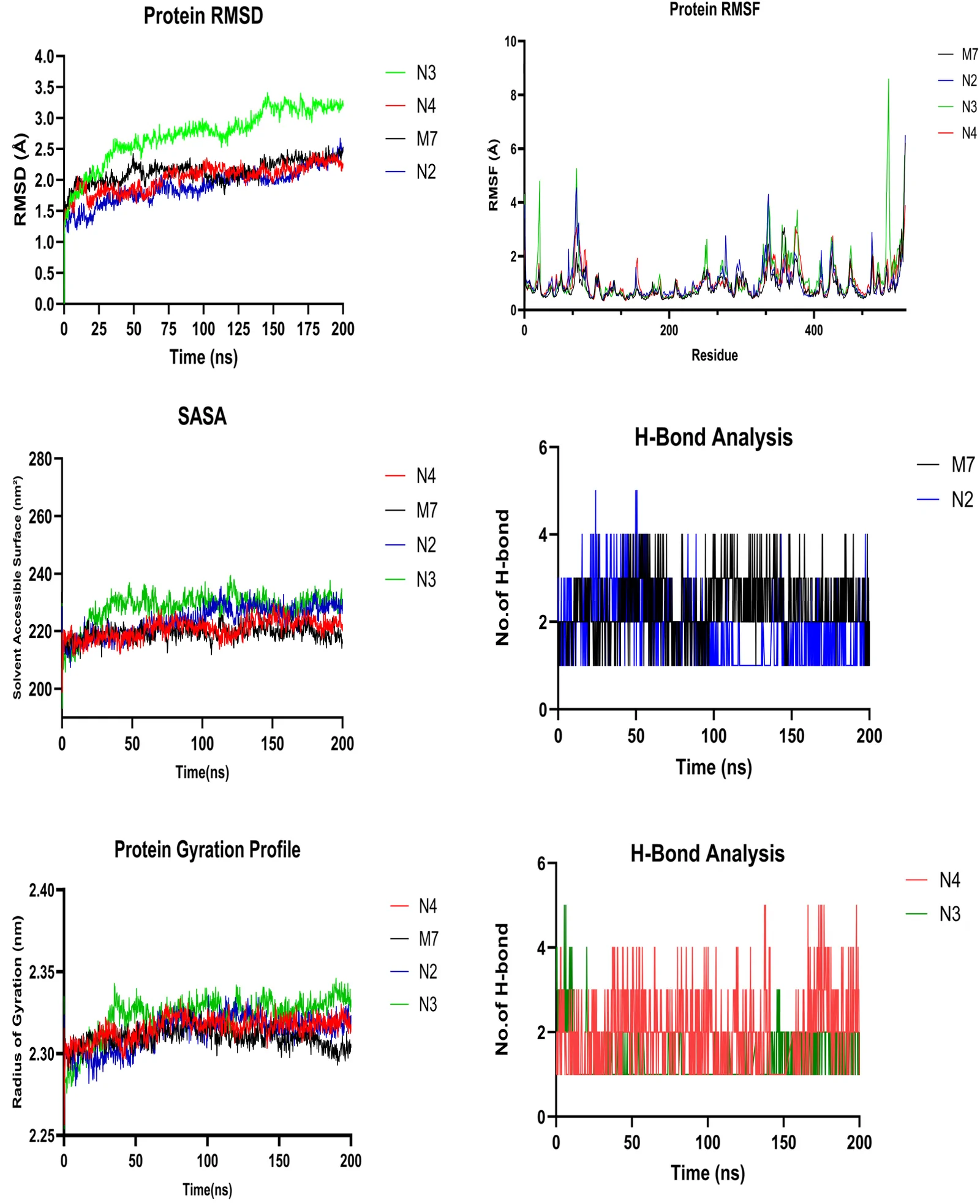
Molecular dynamics profiles of the TcAChE (PDB ID: 2CKM) complexes with M7, N2, N3, and N4 over 200 ns, including RMSD, RMSF, SASA, hydrogen bonds, and radius of gyration (*R*_g_), illustrating the dynamic behavior and stability of the systems.

The stability of the TcAChE complexes with M7, N2, N3 and N4 was studied by molecular dynamics simulations for 200 ns (Fig. 16). The RMSD profiles show an initial increase corresponding to the equilibration of the system, followed by a stable phase sustained throughout the simulation. The average RMSD values are similar for M7-2CKM, N2-2CKM and N4-2CKM, while N3-2CKM stabilizes at a higher value but without signs of major structural instability. Analysis of RMSF reveals that the majority of residues present low to moderate fluctuations, mainly localized in solvent-exposed regions. The region between residues 520 and 540 shows more pronounced flexibility, with N3-2CKM having higher values than the other systems. However, these fluctuations remain localized and do not affect the overall structural stability of the protein. The radius of gyration is relatively constant for all the systems, indicating preserved compactness and no evidence of unfolding. Similarly, SASA profiles show moderate fluctuations without drastic changes, indicating relatively stable solvent exposure during the simulation.

Analysis of hydrogen bonds indicates that all the complexes preserve their ligand–protein interactions during the simulation. The number of hydrogen bonds in M7-2CKM and N2-2CKM varies from 1 to 4 with occasional increases. The hydrogen-bond network of N4-2CKM is more persistent, often displaying two to four interactions. In contrast, N3-2CKM has fewer hydrogen bonds, typically one or two, with more intermittent contacts, although at least one contact is preserved during the simulation. Overall, the results indicate stable dynamic behavior for all complexes. In general, the N2-2CKM and N4-2CKM profiles are similar to the reference system, while N3-2CKM shows relatively higher local flexibility and less persistent hydrogen-bond interactions. However, it remains within a stable dynamic behavior throughout the simulation.

**Fig. 17:**
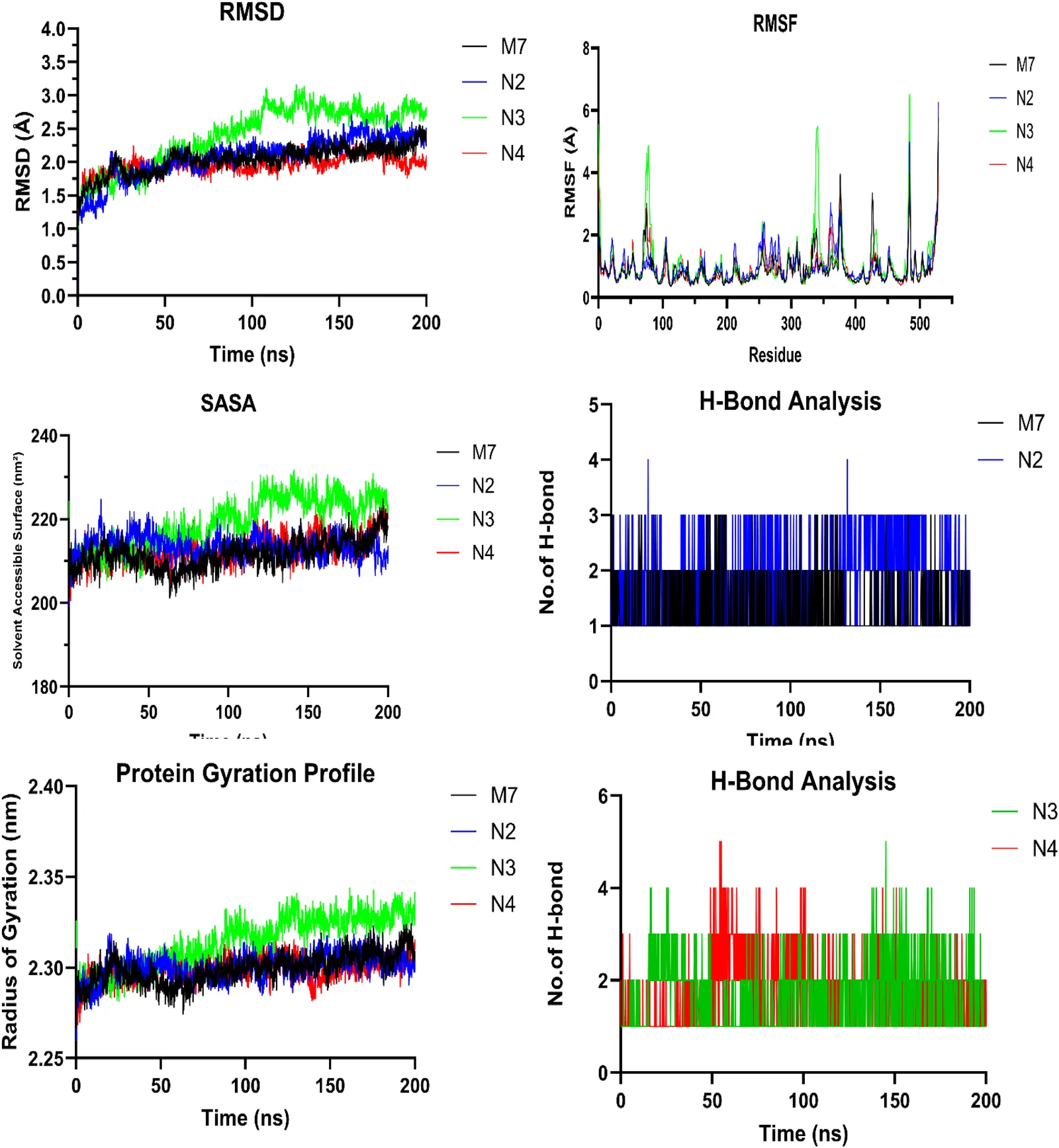
Molecular dynamics profiles of the hAChE (PDB ID: 4EY7) complexes with M7, N2, N3, and N4 over 200 ns, including RMSD, RMSF, SASA, hydrogen bonds, and radius of gyration (*R*_g_), illustrating the dynamic behavior and stability of the systems.

The stability of the human acetylcholinesterase complexes with M7, N2, N3 and N4 was also studied by molecular dynamics simulations for 200 ns (Fig. 17). The RMSD profiles show an initial increase corresponding to system adjustment, followed by a relatively stable phase throughout the simulation. The RMSD values are similar for M7-4EY7, N2-4EY7 and N4-4EY7, whereas N3-4EY7 progressively reaches higher values after approximately 80–100 ns and then stabilizes around a plateau without showing continuous structural drift. RMSF analysis reveals that the majority of residues present low to moderate fluctuations. N3-4EY7 exhibits higher fluctuations than the other systems, but these remain localized and do not indicate global protein instability. The radius of gyration remains relatively constant for all systems, indicating preservation of the overall protein compactness. N3-4EY7 nevertheless displays slightly higher values than the other complexes during the second half of the simulation. Similarly, the SASA profiles show moderate fluctuations without abrupt changes. N3-4EY7 exhibits a slightly higher solvent-accessible surface area, whereas M7-4EY7, N2-4EY7 and N4-4EY7 display relatively similar profiles.

Hydrogen-bond analysis indicates that all four complexes maintain ligand–protein interactions during the simulation. M7-4EY7 generally exhibits one to three hydrogen bonds. N2-4EY7 mainly maintains three hydrogen bonds with occasional transient increases up to four interactions. N3-4EY7 and N4-4EY7 exhibit between one and five hydrogen bonds throughout the trajectory. Overall, the four complexes show stable dynamic behavior. M7-4EY7, N2-4EY7 and N4-4EY7 display relatively similar profiles, whereas N3-4EY7 shows slightly greater flexibility while maintaining stable dynamic behavior throughout the 200 ns simulation.

**Fig. 18:**
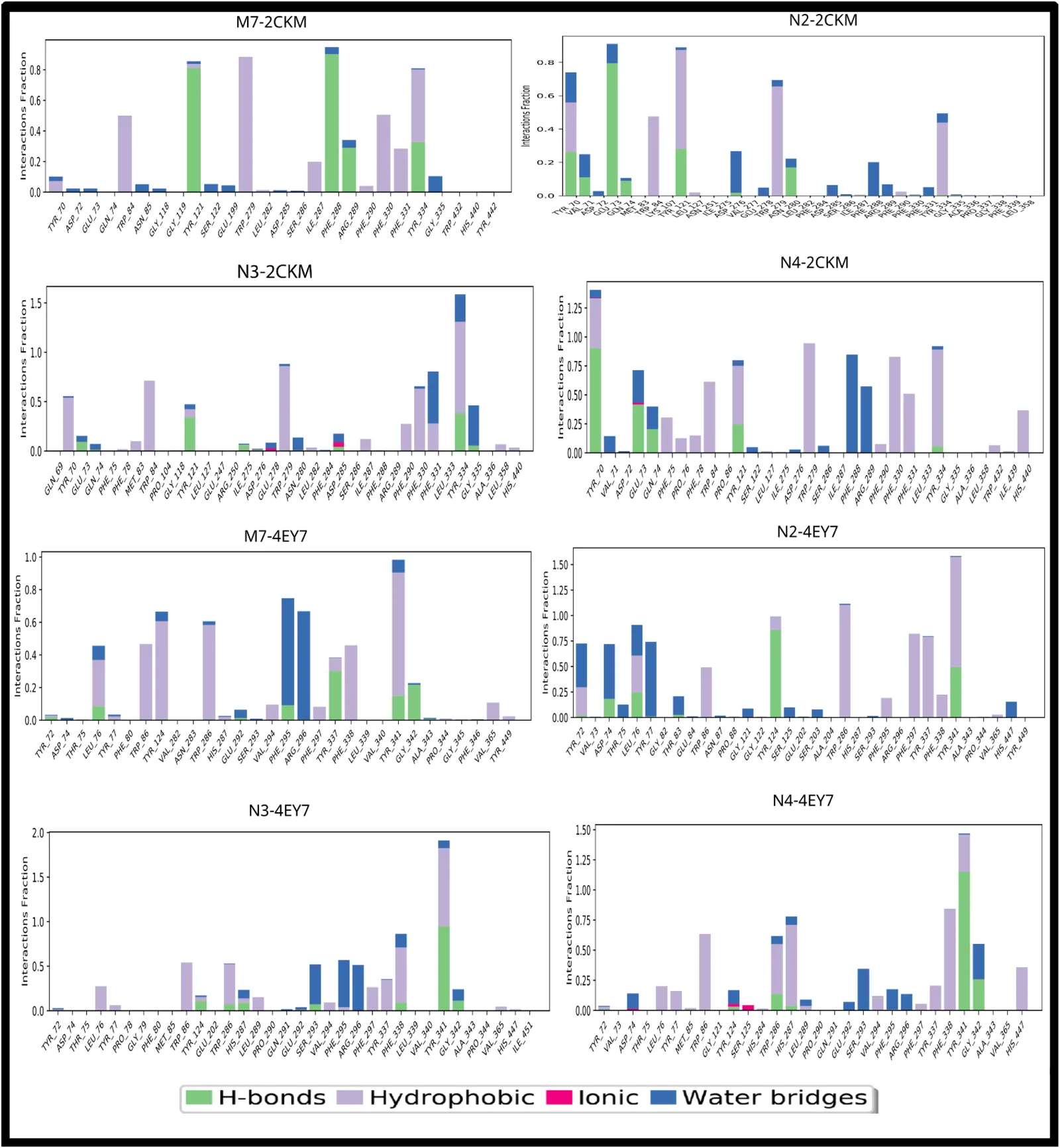
Protein–ligand interaction profiles of M7, N2, N3, and N4 complexes with TcAChE (2CKM) and hAChE (4EY7) during 200 ns MD simulations.

Protein–ligand interaction analysis showed that all four ligands maintained contacts with several important residues of the active-site gorge throughout the 200 ns simulations (Fig. 18). For 2CKM, M7 mainly formed hydrogen bonds with Tyr121, Phe288, Arg289 and Tyr334, hydrophobic interactions with Trp84, Trp279 and Tyr334 and a water bridge with Gly335. N2 maintained hydrogen bonds with Tyr70, Glu73, Tyr121 and Asn280, hydrophobic contacts with Tyr70.

Tyr121 and Trp279 and a water bridge with Phe288. N3 mainly preserved hydrogen bonds with Tyr70, Tyr121 and Tyr334 together with interactions involving Trp84, Trp279 and Phe330. N4 displayed several hydrogen bonds with Tyr70, Glu73, Gln74, Tyr121 and Tyr334 together with hydrophobic interactions involving Trp84, Trp279 and Phe330.

For 4EY7, M7 maintained hydrogen bonds with Tyr337, Tyr341 and Gly342, hydrophobic interactions with Trp86, Tyr124 and Trp286 and water bridges with Phe295 and Arg296. N2 formed hydrogen bonds with Asp74, Leu76, Tyr124 and Tyr341 and hydrophobic interactions with Trp86, Trp286 and Phe297. N3 and N4 also maintained persistent interactions mainly involving Tyr341. Overall, Tyr334 in 2CKM and Tyr341 in 4EY7 appeared as recurrent residues in ligand–protein interactions. In both proteins, the ligands maintained persistent hydrogen-bond and hydrophobic interactions throughout the simulation. Water bridges also participated in the interaction networks of several complexes. These profiles therefore show the maintenance of ligand–protein contacts with both 2CKM and 4EY7 throughout the 200 ns simulations.

**Fig. 19:**
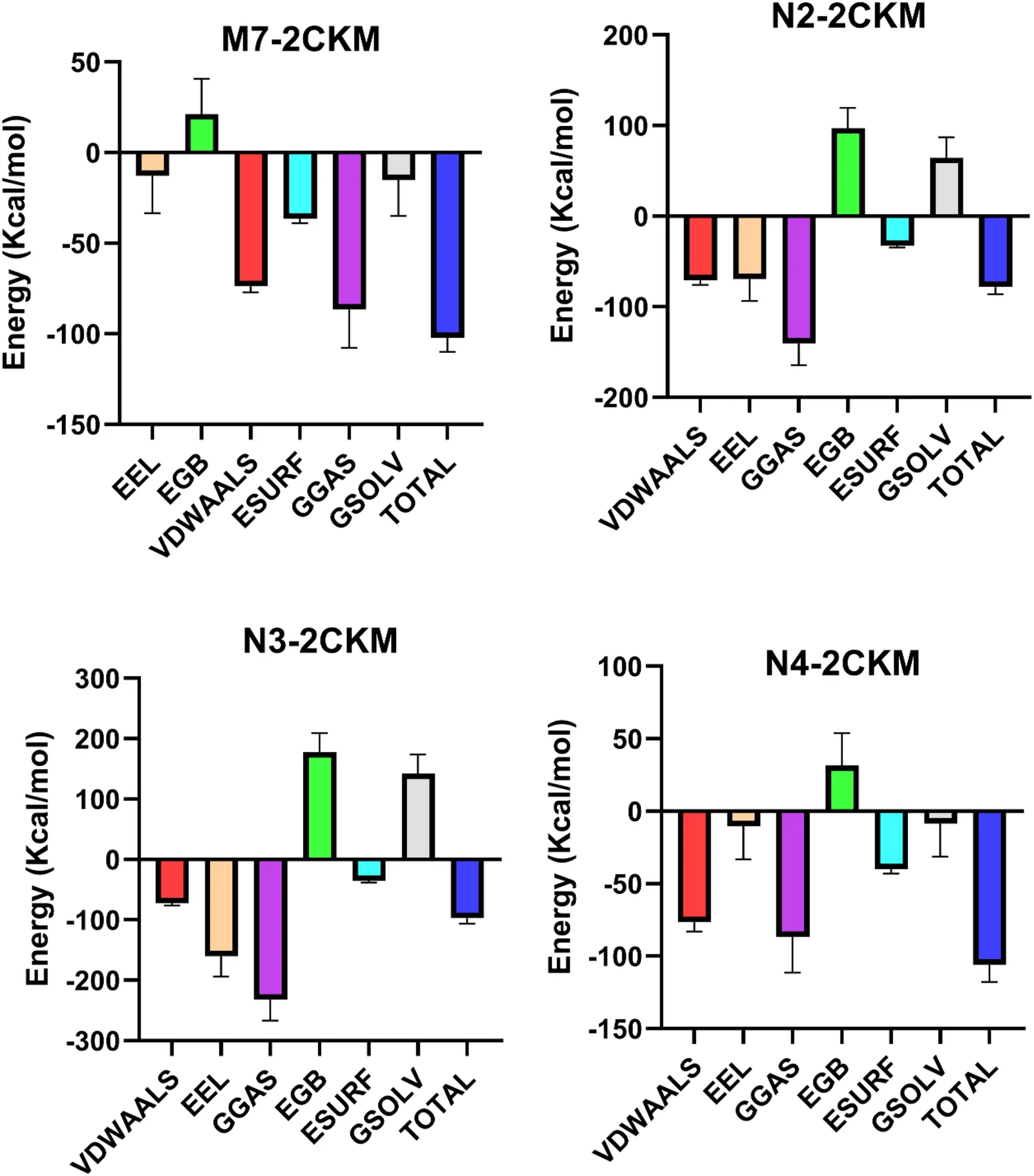
Graphical representation of MM/GBSA energy components of ligand-2CKM complexes.

The stability of the ligand–protein complexes with 2CKM was further evaluated by estimating the binding free energies using the MM-GBSA approach (Table 13 and Fig. 19). All complexes exhibited negative Δ*G*_bind_ values. Among them, N4-2CKM had the most favorable binding free energy at −105.98 ± 11.92 kcal mol^−1^, followed by M7-2CKM at −102.33 ± 7.63 kcal mol^−1^, N3-2CKM at −97.46 ± 8.54 kcal mol^−1^ and N2-2CKM at −78.13 ± 8.18 kcal mol^−1^. The energy decomposition analysis showed that van der Waals interactions were a major favorable contribution to ligand binding in all 2CKM complexes. However, electrostatic contributions varied considerably among the ligands. N3-2CKM showed the strongest favorable electrostatic interaction, which was partially offset by a large polar solvation penalty. In contrast, the non-polar solvation contribution was relatively similar for the four systems, indicating a comparable contribution from the solvent-accessible surface. These results highlight the important role of van der Waals interactions in the stabilization of the ligand–protein complexes and the influence of the balance between electrostatic and solvation contributions on the final binding free energy.

**Table 13:** MM-GBSA binding free energy decomposition of ligand-2CKM complexes (kcal mol^−1^)^a^

		Ligand-receptor			
		M7-2CKM	N2-2CKM	N3-2CKM	N4-2CKM
Energy (Kcal mol^−1^)	VDWAALS	−73.83 ± 3.31	−70.98 ± 4.76	−72.27 ± 4.03	−76.50 ± 6.55
	EEL	−12.76 ± 20.58	−69.54 ± 23.92	−160.16 ± 33.63	−10.26 ± 23.00
	GGAS	−86.59 ± 21.20	−140.52 ± 23.90	−232.44 ± 34.27	−86.76 ± 24.66
	EGB	21.31 ± 19.48	97.04 ± 22.37	177.70 ± 31.67	31.49 ± 22.38
	ESURF	−36.41 ± 2.48	−32.52 ± 2.02	−35.32 ± 2.78	−39.94 ± 2.98
	GSOLV	−15.10 ± 19.77	64.52 ± 22.74	142.39 ± 31.51	−8.45 ± 22.85
	TOTAL (Δ*G*_bind_)	−102.33 ± 7.63	−78.13 ± 8.18	−97.46 ± 8.54	−105.98 ± 11.92

a Values are expressed as mean ± standard deviation in kcal mol^−1^.

**Fig. 20:**
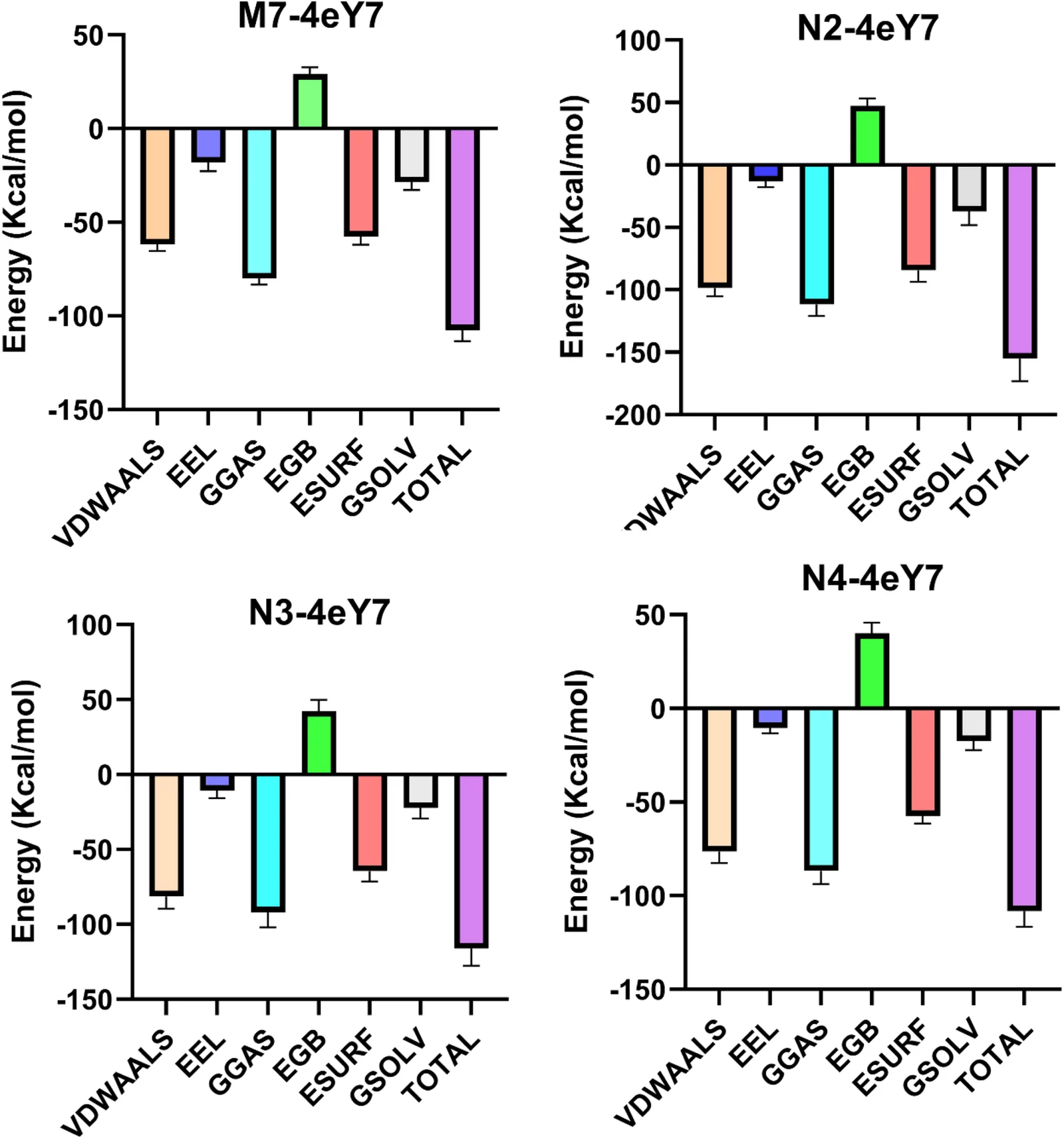
Graphical representation of MM/GBSA energy components of ligand-4EY7 complexes.

The binding free energies of the ligand-4EY7 complexes were then evaluated using the MM-GBSA approach (Table 14 and Fig. 20). All complexes exhibited negative Δ*G*_bind_ values. N2-4EY7 showed the most favorable binding free energy at −155.22 ± 17.89 kcal mol^−1^, followed by N3-4EY7 at −116.11 ± 11.46 kcal mol^−1^, N4-4EY7 at −108.31 ± 8.31 kcal mol^−1^ and M7-4EY7 at −107.55 ± 5.83 kcal mol^−1^. N4-4EY7 and M7-4EY7 therefore displayed very similar values. As shown in Fig. 20, van der Waals interactions contributed favorably to binding in all four complexes, with the strongest contribution observed for N2-4EY7 followed by N3-4EY7. M7-4EY7 showed the most favorable electrostatic contribution, whereas N2-4EY7 displayed the most negative gas-phase energy. The polar solvation contribution EGB was positive for all four complexes, whereas the non-polar ESURF contribution was negative. The combination of these two terms resulted in negative GSOLV values for all complexes. Overall, the MM-GBSA analysis showed favorable energetic profiles for all four systems, with N2-4EY7 exhibiting the most favorable Δ*G*_bind_ value.

**Table 14:** MM-GBSA binding free energy decomposition of ligand-4EY7 complexes (kcal mol^−1^)^a^

		Ligand-receptor			
		M7-4EY7	N2-4EY7	N3-4EY7	N4-4EY7
Energy (Kcal mol^−1^)	VDWAALS	−61.83 ± 3.41	−98.58 ± 6.54	−81.30 ± 8.15	−76.34 ± 6.31
	EEL	−18.01 ± 4.68	−13.01 ± 4.55	−10.70 ± 4.99	−10.48 ± 2.78
	GGAS	−79.85 ± 3.46	−111.59 ± 9.31	−92.00 ± 9.89	−86.82 ± 6.97
	EGB	29.05 ± 3.67	47.35 ± 6.07	42.30 ± 7.49	40.07 ± 5.76
	ESURF	−57.62 ± 4.26	−84.43 ± 9.25	−64.44 ± 6.88	−57.41 ± 3.95
	GSOLV	−28.57 ± 4.14	−37.08 ± 11.10	−22.14 ± 7.04	−17.34 ± 4.92
	TOTAL (Δ*G*_bind_)	−107.55 ± 5.83	−155.22 ± 17.89	−116.11 ± 11.46	−108.31 ± 8.31

a Values are expressed as mean ± standard deviation in kcal mol^−1^.

**Fig. 21:**
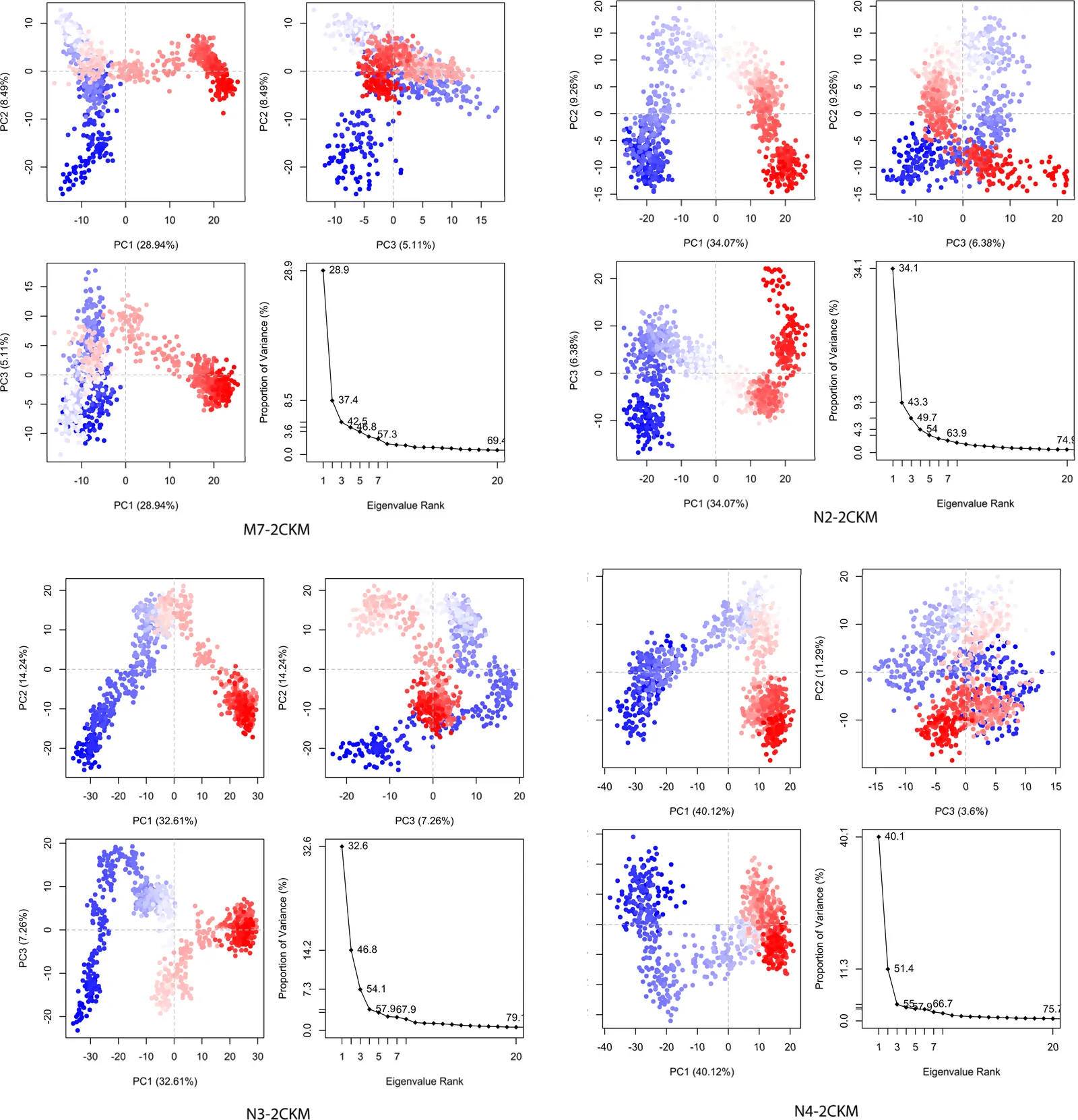
Principal component analysis (PCA) of ligand-2CKM complexes showing conformational space and eigenvalue distribution.

Principal component analysis PCA was used to characterize the essential collective motions of the ligand-2CKM complexes (Fig. 21 and Table 15). The PCA projections show that all ligand-2CKM complexes sampled well-defined conformational spaces over the 200 ns simulation, indicating stable global dynamics. The M7-2CKM complex showed a structured distribution of conformations with the first principal component PC1 explaining 28.94% of the variance. Similarly, the N2-2CKM and N3-2CKM complexes presented similar conformational distributions with PC1 explaining 34.07% and 32.61% of the total variance, respectively. The N4-2CKM complex also preserved a well-defined conformational distribution, with PC1 accounting for the highest percentage of total variance at 40.12%. Overall, the PCA results indicate that the four complexes remained in a dynamically stable state throughout the simulation, with only small differences in conformational sampling.

**Table 15:** Percentage of variance explained by the first three principal components (PC1–PC3) of ligand-2CKM complexes

Complex	PC1	PC2	PC3
M7-2CKM	28.94%	8.49%	5.11%
N2-2CKM	34.07%	9.26%	6.38%
N3-2CKM	32.61%	14.24%	7.26%
N4-2CKM	40.12%	11.29%	3.60%

**Fig. 22:**
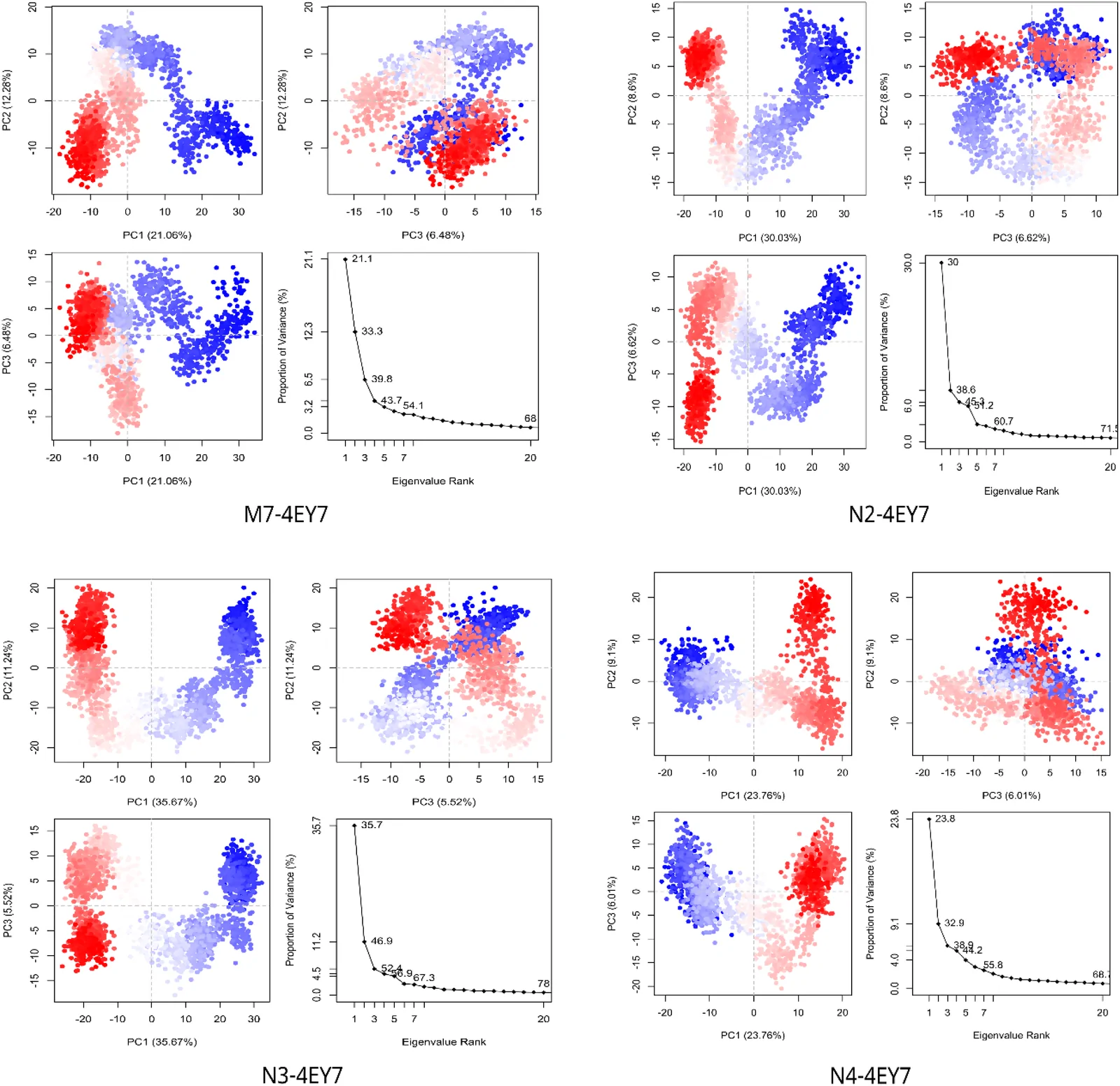
Principal component analysis (PCA) of ligand-4EY7 complexes showing conformational space and eigenvalue distribution.

Principal component analysis PCA was also used to characterize the essential collective motions of the ligand-4EY7 complexes (Fig. 22 and Table 16). The PCA projections showed that all four complexes sampled well-defined conformational spaces throughout the 200 ns simulation. The M7-4EY7 complex displayed a structured conformational distribution with PC1 explaining 21.06% of the total variance. Similarly, N2-4EY7 and N4-4EY7 showed defined conformational distributions with PC1 accounting for 30.03% and 23.76% of the total variance, respectively. N3-4EY7 exhibited the highest PC1 contribution with 35.67% of the total variance. Overall, the PCA results showed that the four complexes maintained organized conformational behavior throughout the simulation, with moderate differences in the amplitude of their collective motions.

**Table 16:** Percentage of variance explained by the first three principal components (PC1–PC3) of ligand-4EY7 complexes

Complex	PC1	PC2	PC3
M7–4EY7	21.06%	12.28%	6.48%
N2–4EY7	30.03%	8.60%	6.62%
N3–4EY7	35.67%	11.24%	5.62%
N4–4EY7	23.76%	9.10%	6.01%

**Fig. 23:**
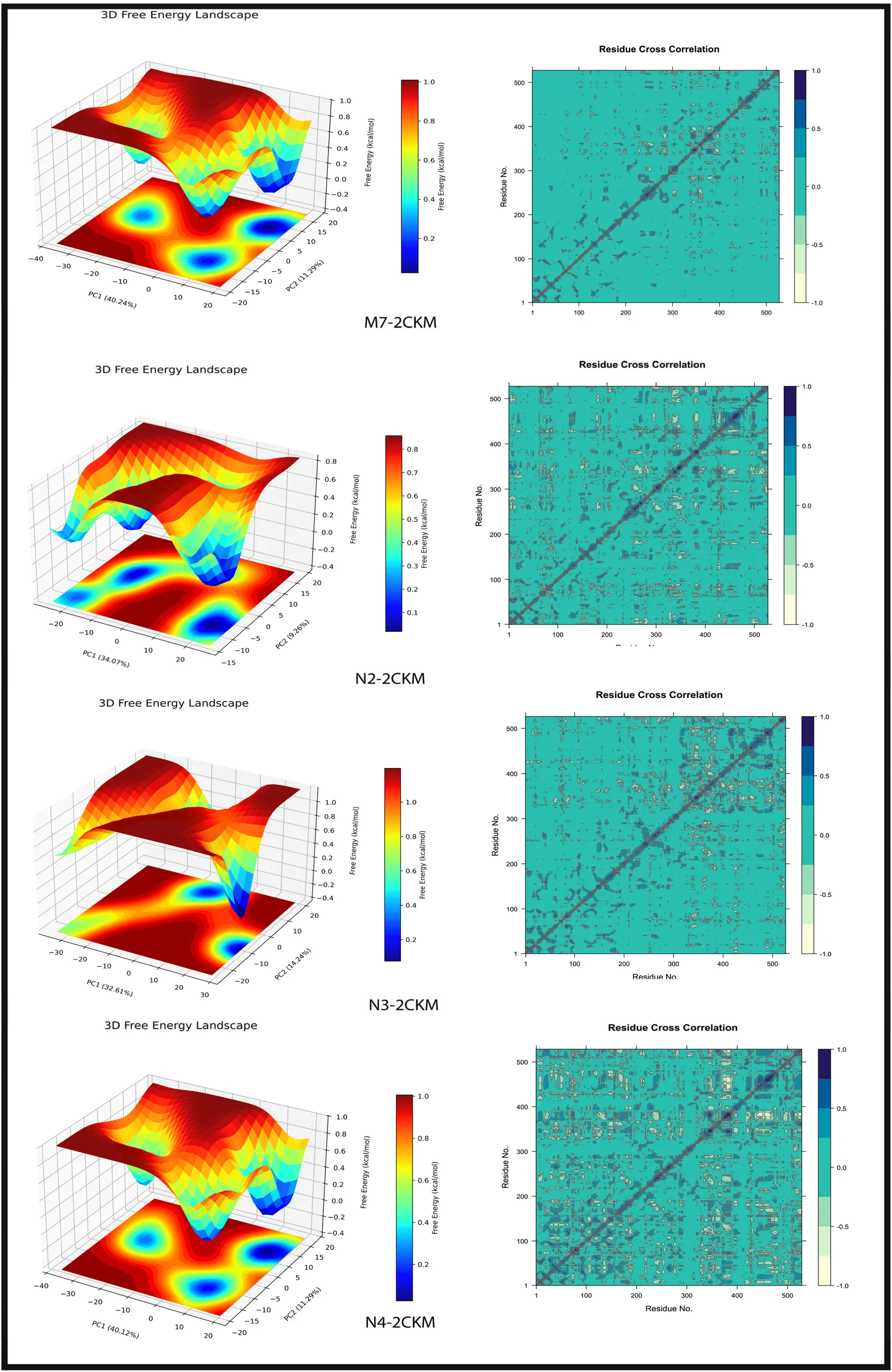
Free energy landscape (FEL) and dynamic cross-correlation matrix (DCCM) analyses of ligand-2CKM complexes.

To further examine the conformational stability of the ligand-2CKM complexes, free energy landscapes FEL were constructed using the first two principal components as reaction coordinates (Fig. 23). All complexes showed well-defined low-energy minima, indicating that energetically favorable conformational states were achieved during the simulation. Small variations were observed in the distribution and shape of the energy minima among the complexes, but no major conformational transitions toward high-energy or strongly perturbed states were observed. These results indicate that the four ligand-2CKM complexes maintained favorable conformational states throughout the simulation.

DCCM analysis of the ligand-2CKM complexes (Fig. 23) showed that the overall patterns of correlated and anticorrelated residue motions were mostly preserved in all four complexes. M7-2CKM showed a relatively homogeneous correlation profile, whereas N2-2CKM displayed a mild increase in local anticorrelated motions. The correlated regions were slightly more pronounced for N3-2CKM and N4-2CKM, particularly for N4-2CKM, without altering the overall dynamic organization. These findings suggest that the intrinsic collective motions of TcAChE were not strongly perturbed by ligand binding during the simulation.

**Fig. 24:**
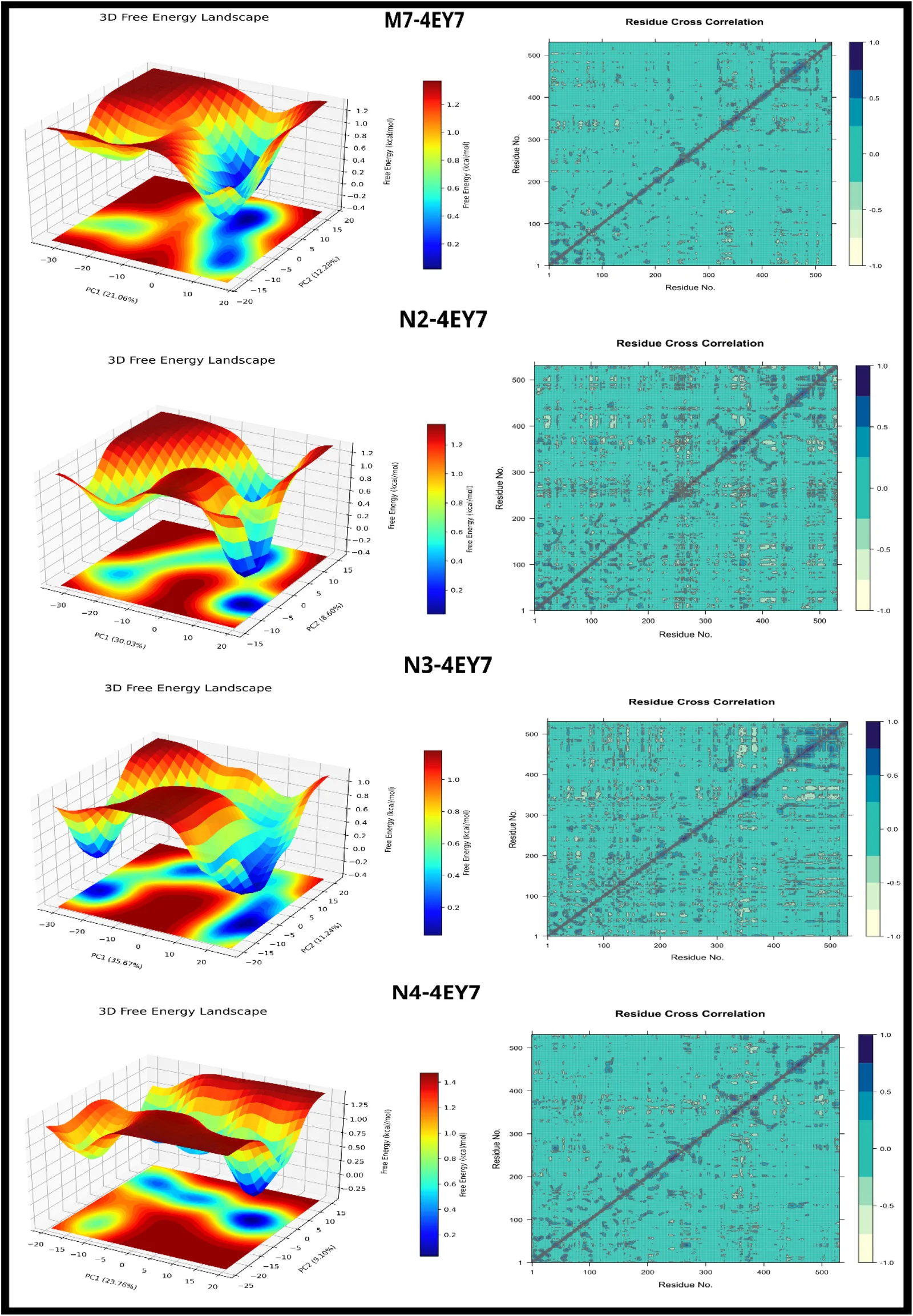
Free energy landscape (FEL) and dynamic cross-correlation matrix (DCCM) analyses of ligand-4EY7 complexes.

To further examine the conformational behavior of the ligand-4EY7 complexes, free energy landscapes FEL were constructed using the first two principal components (Fig. 24). All four complexes displayed well-defined low-energy regions, indicating the sampling of energetically favorable conformational states throughout the 200 ns simulation. M7-4EY7 and N2-4EY7 showed relatively well-defined energy basins, whereas N3-4EY7 displayed a broader distribution with several low-energy regions. N4-4EY7 also exhibited several accessible energetic regions with a clearly identifiable main basin. Despite these differences in the distribution of the minima, none of the complexes showed a landscape dominated exclusively by high-energy regions, indicating that favorable conformational states were maintained during the simulation.

DCCM analysis (Fig. 24) showed that the four complexes maintained overall patterns of correlated and anticorrelated residue motions. M7-4EY7 displayed a relatively homogeneous profile with limited local variations. N2-4EY7 and N3-4EY7 showed slightly more pronounced regions of correlation and anticorrelation, indicating local differences in collective residue motions. N4-4EY7 also maintained a well-defined overall profile with some localized variations. Overall, the FEL and DCCM results showed that all four complexes sampled energetically favorable states while maintaining an organized global dynamic behavior throughout the 200 ns simulation.

The MM-GBSA ranking differed from the docking ranking for both AChE structures because the two approaches evaluate different aspects of ligand–protein binding. Docking scores are based on static poses and empirical scoring functions, whereas MM-GBSA incorporates interaction energies and solvation contributions calculated from conformations sampled during the MD simulations. For 2CKM, N2-2CKM showed the most favorable docking energy, whereas N4-2CKM exhibited the most favorable MM-GBSA binding free energy. The favorable gas-phase interaction energy of N2-2CKM was substantially offset by a high polar solvation penalty, particularly the positive EGB contribution, whereas N4-2CKM showed a more favorable balance between van der Waals, electrostatic and solvation contributions. For 4EY7, N4-4EY7 showed the most favorable docking energy, whereas N2-4EY7 exhibited the most favorable MM-GBSA Δ*G*_bind_ value. This difference was associated with the particularly favorable van der Waals contribution and more negative gas-phase energy of N2-4EY7. These results show that docking and MM-GBSA rankings are not directly equivalent and reflect complementary aspects of ligand–protein binding.

In summary, the molecular dynamics results were complemented by MM-GBSA, PCA, FEL and DCCM analyses, which provide complementary information on ligand binding and the conformational behavior of the complexes. All four ligands generally maintained dynamically stable complexes with both TcAChE and hAChE throughout the 200 ns simulations, while N3 displayed relatively greater flexibility in both systems. The interaction profiles also confirmed persistent contacts with important residues of the active-site gorge in both structures. The differences observed in the MM-GBSA results indicate that the energetic contributions of the ligands vary according to the structural environment of AChE. Taken together, these analyses demonstrate an overall favorable dynamic behavior of the investigated compounds with both AChE structures.

## 4. Conclusion

*In silico* approaches were used to investigate the inhibitory potential of tacrine-based carbamate derivatives against acetylcholinesterase. The 3D-QSAR models (CoMFA and CoMSIA) exhibited satisfactory predictive performance, as supported by the validation procedures and the consistent results obtained from the complementary 2D-QSAR models. The analyses highlighted the importance of steric effects and hydrogen-bond donor interactions in modulating biological activity. The designed derivatives displayed favorable predicted ADMET profiles, while DFT calculations characterized their electronic properties. Molecular docking against TcAChE (2CKM) and hAChE (4EY7) indicated strong interactions within the AChE active sites, while molecular dynamics simulations supported the stable behavior of the selected complexes with both AChE structures over 200 ns. In addition, the MM-GBSA, PCA, FEL, and DCCM analyses provided further support for favorable binding energetics, stable conformational behavior, and coordinated collective motions. Taken together, these results support the use of the proposed tacrine-modified carbamate derivatives as promising candidates for further investigation. However, the predicted activities of N1–N4 should be considered as computational estimates, and their synthesis and experimental biological evaluation are required to confirm their actual inhibitory activities.

## Author contributions

Hajar El Ouarti: conceptualization, methodology, software, formal analysis, investigation, data curation, visualization, writing – original draft. Abdelilah Toughzaoui: supervision, methodology, validation, project administration, writing – review & editing. Kamal Laourdani: validation, formal analysis, writing – review & editing. Abderrafie Drajou: validation, writing – review & editing. Abdeslam Dakhama: validation, writing – review & editing. Usama Raza: Molecular dynamics simulations, principal component analysis, formal analysis, writing – review & editing. Mohammed Bensaka: Biological expertise, validation, writing – review & editing. Lamy M. Mohamed Hamed: funding acquisition, resources, writing – review & editing, supervision. Khalil Azzaoui: conceptualization, methodology, writing – review & editing, funding acquisition, supervision. Hammouti Belkheir: validation, resources, writing – review & editing. Ahmed El-Harairy: methodology, software, validation, formal analysis, writing – review & editing, visualization, supervision. Kamal Moradi: supervision, validation, writing – review & editing. Abdelkrim Ouammou: conceptualization, supervision, project administration, writing – review & editing.

## Conflicts of interest

There are no conflicts to declare.

## Data Availability

The data presented in this study are available upon request from the corresponding author.
